# Ndel1 disfavors dynein–dynactin–adaptor complex formation in two distinct ways

**DOI:** 10.1016/j.jbc.2023.104735

**Published:** 2023-04-21

**Authors:** Sharon R. Garrott, John P. Gillies, Aravintha Siva, Saffron R. Little, Rita El Jbeily, Morgan E. DeSantis

**Affiliations:** 1 Department of Molecular, Cellular, and Developmental Biology, University of Michigan, Ann Arbor, Michigan, USA; 2 Department of Biological Chemistry, University of Michigan, Ann Arbor, Michigan, USA; 3 Program in Chemical Biology, University of Michigan, Ann Arbor, Michigan, USA

**Keywords:** cytoplasmic dynein-1, microtubules, Lis1, Ndel1, Nde1, dynactin

## Abstract

Dynein is the primary minus-end-directed microtubule motor protein. To achieve activation, dynein binds to the dynactin complex and an adaptor to form the “activated dynein complex.” The protein Lis1 aids activation by binding to dynein and promoting its association with dynactin and the adaptor. Ndel1 and its paralog Nde1 are dynein- and Lis1-binding proteins that help control dynein localization within the cell. Cell-based assays suggest that Ndel1–Nde1 also work with Lis1 to promote dynein activation, although the underlying mechanism is unclear. Using purified proteins and quantitative binding assays, here we found that the C-terminal region of Ndel1 contributes to dynein binding and negatively regulates binding to Lis1. Using single-molecule imaging and protein biochemistry, we observed that Ndel1 inhibits dynein activation in two distinct ways. First, Ndel1 disfavors the formation of the activated dynein complex. We found that phosphomimetic mutations in the C-terminal domain of Ndel1 increase its ability to inhibit dynein–dynactin–adaptor complex formation. Second, we observed that Ndel1 interacts with dynein and Lis1 simultaneously and sequesters Lis1 away from its dynein-binding site. In doing this, Ndel1 prevents Lis1-mediated dynein activation. Together, our work suggests that *in vitro*, Ndel1 is a negative regulator of dynein activation, which contrasts with cellular studies where Ndel1 promotes dynein activity. To reconcile our findings with previous work, we posit that Ndel1 functions to scaffold dynein and Lis1 together while keeping dynein in an inhibited state. We speculate that Ndel1 release can be triggered in cellular settings to allow for timed dynein activation.

Cytoplasmic dynein-1 (dynein) is a microtubule-associated molecular motor that is responsible for nearly all minus-end-directed force generation in most eukaryotes (1). Dynein traffics hundreds of unique types of cargos, positions the centrosome, facilitates spindle focusing and alignment during mitosis, and strips spindle assembly checkpoint components present at the kinetochore to promote metaphase–anaphase transition (1, 2, 3, 4, 5). Mutations in dynein or its regulatory partners are associated with a host of neurodevelopmental and neurodegenerative diseases (6).

Dynein is a large protein complex comprised of six different subunits, each present in two copies (Figs. 1*A* and S1*A*). The largest subunit, called the heavy chain, contains a motor domain that is a ring of six AAA (ATPase Associated with various cellular Activities) domains (Fig. S1*A*). AAA1, AAA3, and AAA4 are active ATPase modules (7, 8). The heavy chain also contains dynein’s microtubule-binding domain and a large projection called the tail that acts as a platform for the assembly of the other subunits, including the intermediate chains, the light intermediate chains, and three different light chains (Figs. 1*A* and S1*A*) (7, 8). In the absence of other protein factors, dynein exists in an autoinhibited conformation (called “Phi”) and cannot engage productively with the microtubule track (Fig. S1*A*) (9). Dynein is activated by binding to the multisubunit protein complex, dynactin, and one of a family of activating adaptor proteins (adaptors) (Fig. S1*A*) (10, 11). This complex, which we will call the “activated dynein complex,” positions the dynein motor domains in a parallel conformation that is competent to move processively along the microtubule (Fig. S1*A*) (12, 13, 14, 15). In addition to assembling into a complex to activate dynein motility, adaptors also link dynein to cargo (1). Another regulatory protein, Lis1, promotes formation of the activated dynein complex by binding and converting Phi into a conformation that is primed to bind dynactin and adaptor (Fig. S1*A*) (16, 17, 18, 19, 20, 21, 22, 23, 24). Together, dynactin, adaptors, and Lis1 are three of four of dynein’s core regulatory machinery.

**Figure 1:**
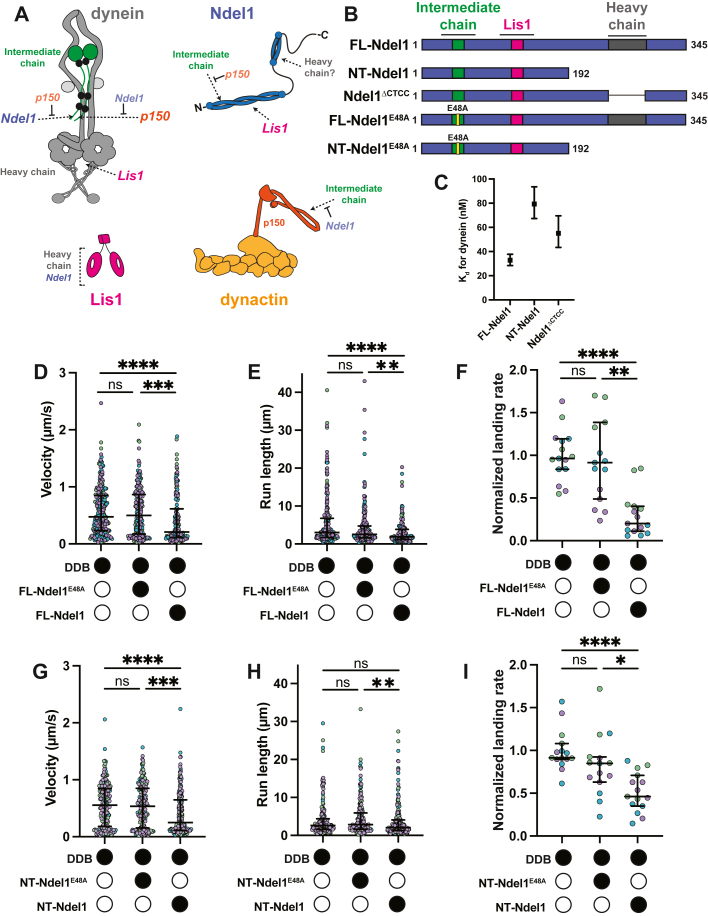
**Ndel1 reduces motility of DDB complexes.***A*, diagrams of dynein and its regulators. *Dashed arrows* indicate binding sites, and *blunted arrows* indicate competitive binding. Dynein heavy chain (*gray*) and intermediate chain (*green*) are labeled. Light intermediate chains (*light gray*) and light chains (*black*) are unlabeled. Ndel1 is shown in *blue*, Lis1 is shown in *pink*, and dynactin in shades of *orange*. *B*, schematics of Ndel1 constructs. *C*, *K*_*d*_s between dynein and Ndel1 constructs. Error bars indicate 95% confidence intervals. *D*, single-molecule velocity of DDB complexes in the absence *(white circles*) or the presence (*black circles*) of 300 nM FL-Ndel1^E48A^ or FL-Ndel1. n = 257 (no Ndel1); 209 (FL-Ndel1^E48A^); 181 (FL-Ndel1). Error bars are median  ±  interquartile range. Statistical analysis was performed using a Kruskal–Wallis with Dunn’s multiple comparisons test. *p* Values: ns > 0.9999; ∗∗∗0.0001; ∗∗∗∗ < 0.0001. *E*, single-molecule run lengths of DDB complexes in the absence (*white circles*) or the presence (*black circles*) of 300 nM FL-Ndel1^E48A^ or FL-Ndel1. n = 257 (no Ndel1); 209 (FL-Ndel1^E48A^); 181 (FL-Ndel1). Error bars are median  ±  interquartile range. Statistical analysis was performed using a Kruskal–Wallis with Dunn’s multiple comparisons test. *p* Values: ns = 0.1193; ∗∗0.0082; ∗∗∗∗ < 0.0001. *F*, single-molecule events per micrometer of microtubule per nanometer dynein for DDB complexes in the absence (*white circles*) or the presence (*black circles*) of 300 nM FL-Ndel1^E48A^ or FL-Ndel1. Data are normalized to the no Ndel1 control for each replicate. n = 15 microtubules per condition. Error bars are median  ±  interquartile range. Statistical analysis was performed using a Brown–Forsythe and Welch ANOVA with Dunnet’s T3 multiple comparisons test. *p* Values: ns >0.9999; ∗∗0.0012; ∗∗∗∗ < 0.0001. *G*, single-molecule velocity of DDB complexes in the absence (*white circles*) or the presence (*black circles*) of 300 nM NT-Ndel1^E48A^ or NT-Ndel1. n = 193 (no Ndel1); 207 (NT-Ndel1^E48A^); 215 (NT-Ndel1). Error bars are median  ±  interquartile range. Statistical analysis was performed using a Kruskal–Wallis with Dunn’s multiple comparisons test. *p* Values: ns >0.9999; ∗∗∗0.0005; ∗∗∗∗ < 0.0001. *H*, single-molecule run lengths of DDB complexes in the absence (*white circles*) or the presence (*black circles*) of 300 nM NT-Ndel1^E48A^ or NT-Ndel1. n = 193 (no Ndel1); 207 (NT-Ndel1^E48A^); 215 (NT-Ndel1). Error bars are median  ±  interquartile range. Statistical analysis was performed using a Kruskal–Wallis with Dunn’s multiple comparisons test. *p* Values: ns (no Ndel1 *versus* NT-Ndel1^E48A^) = 0.8699; ns (no Ndel1 *versus* NT-Ndel1) = 0.0960; ∗∗0.0032. *I*, single-molecule events per micrometer of microtubule per nanometer dynein for DDB complexes in the absence (*white circles*) or the presence (*black circles*) of 300 nM NT-Ndel1^E48A^ or NT-Ndel1. Data are normalized to the no Ndel1 control for each replicate. n = 15 microtubules per condition. Error bars are median  ±  interquartile range. Statistical analysis was performed using a Brown–Forsythe and Welch ANOVA with Dunnet’s T3 multiple comparisons test. *p* Values: ns = 0.3462; ∗0.0261; ∗∗∗∗ < 0.0001. DDB, dynein–dynactin–BicD2; FL, full length.

The fourth member of dynein’s core regulatory partners is the protein Ndel1 and its paralog, Nde1. Although there is no structure of full-length (FL) Ndel1 or Nde1, structures of the N-terminal half of Ndel1 reveal that it is a long coiled coil (25). The C-terminal half is largely disordered, except for a short stretch of ∼40 amino acids that forms a coiled coil (Fig. S1, *B* and *C*) (26). Many proteins bind to Ndel1–Nde1 to localize dynein throughout the cell cycle. For example, interactions between CENP-F and Ndel1–Nde1 help position dynein at the nuclear pore for centrosome positioning and at the kinetochore, where dynein will eventually traffic checkpoint proteins toward the spindle poles to facilitate the metaphase–anaphase transition (5, 27, 28, 29). Ndel1 and Nde1 may also support dynein localization to cargo in interphase. For example, interactions between Ndel1–Nde1 and Rab9 promote dynein localization to endosomes (30).

In addition to localizing dynein within the cell, there is evidence that Ndel1 and Nde1 directly support dynein activation. In human cells, concurrent depletion of Ndel1 and Nde1 causes Golgi dispersal, whereas inhibition of Ndel1 and Nde1 by a function-blocking antibody causes acidic cargo dispersal (3, 31). Both effects suggest a reduction in dynein-driven minus-end-directed trafficking. Depletion of the Ndel1 homolog in fungi causes defects in nuclear positioning in a manner that is consistent with reduced dynein activity (32, 33, 34, 35). Furthermore, in filamentous fungi, expression of a dynein mutant that cannot assume the Phi structure can partially rescue a defect in dynein-mediated early endosome transport caused by Ndel1 depletion, suggesting that Ndel1 serves to activate dynein (23).

Ndel1 and Nde1 likely work together with Lis1 to support dynein activity. *In vitro* experiments conducted with purified mammalian and yeast proteins show that Ndel1–Nde1 promote Lis1–dynein association (36, 37). In addition, cell-based assays performed in many organisms and cell types show that deleterious effects of knockdown of either protein can be rescued by overexpression of the other, suggesting that Lis1 and Ndel1–Nde1 operate in the same pathway. For example, Ndel1 depletion in filamentous fungi and *Xenopus* extracts results in nuclear distribution defects and spindle focusing defects, respectively. Both phenotypes are rescued by Lis1 overexpression (38, 39, 40). Similarly, in developing fly brains, dendritic arborization defects caused by Nde1 deletion are rescued by Lis1 overexpression (41). Conversely, in mammalian cells, Ndel1 expression rescues spindle orientation defects and Golgi dispersal caused by Lis1 knockdown (3, 42). A prevailing model is that Ndel1–Nde1 tether dynein and Lis1, stabilizing their interaction (36, 38, 43). A prediction from this model is that Ndel1–Nde1 will increase the ability of Lis1 to form the activated dynein complex.

Multiple structural and biochemical studies have refined our understanding of how dynactin, adaptors, and Lis1 interact with dynein to facilitate activation (Fig. S1*A*) (12, 13, 14, 15, 16, 17, 18, 19, 20, 22, 24). The position of Ndel1 and Nde1 in the interaction network of dynein and its regulators is less clear. In addition to binding the beta propeller of Lis1, Ndel1 and Nde1 bind to the dynein intermediate chain and heavy chain (Fig. 1*A*). The first ∼50 amino acids of Ndel1–Nde1 interacts with the disordered N-terminal tail of the intermediate chain (Fig. 1*A*) (37, 38, 43, 44, 45, 46). This interaction has been validated *in vitro* and in cell-based assays. Interestingly, Ndel1–Nde1 bind the intermediate chain competitively with a coiled coil in p150 subunit of dynactin (called CC1) (Fig. 1*A*) (45, 46, 47, 48). The purported heavy chain–binding site of Ndel1, which is less validated, is within the C-terminal coiled coil of Ndel1 (amino acids ∼253–293) (Fig. 1*A*) (49, 50). On the dynein side, this interaction has not been mapped with high confidence but may span AAA1 and the heavy-chain C-terminal tail (50).

We set out to investigate how Ndel1 affects dynein activity and test the model that Ndel1 tethers Lis1 to dynein to promote activation. Using single-molecule imaging and protein biochemistry, we found that Ndel1 disfavors formation of the activated dynein complex, most likely *via* competition with p150 for intermediate chain binding. Despite the C terminus of Ndel1 being dispensable for inhibiting dynein activation, it increases the potency of inhibition. Furthermore, phosphomimetic mutations in the C terminus of Ndel1 increase its ability to inhibit complex formation. Our work also revealed that Ndel1 and Lis1 binding is negatively regulated by the C-terminal half of Ndel1, suggesting that Ndel1 is autoinhibited with respect to Lis1 association. We found that although Ndel1 can bind both Lis1 and dynein simultaneously, Lis1 cannot bind Ndel1 and dynein at the same time. This competition significantly attenuates the ability of Lis1 to promote activated dynein complex assembly in the presence of Ndel1. Our work suggests that Ndel1 has two distinct modes of preventing dynein activation: disfavoring dynein complex assembly by binding the intermediate chain and preventing Lis1-mediated activation.

## Results

### The N-terminal half of Ndel1 inhibits dynein motility

The function of the two dynein-binding sites of Ndel1 has been controversial. To characterize the interaction between dynein and Ndel1, we purified SNAP-tagged, recombinant FL human dynein with all associated accessory chains (dynein), FL-Ndel1, and several Ndel1 mutant or deletion constructs (Figs. 1*B* and Fig. S1*D*). These constructs include a truncation of Ndel1 containing only the N-terminal coiled coil (NT-Ndel1), a well-characterized Ndel1 mutant that disrupts binding to the intermediate chain in both the FL-Ndel1 and NT-Ndel1 backgrounds (FL-Ndel1^E48A^ and NT-Ndel1^E48A^), and a construct with the coiled coil containing the purported heavy chain–binding site deleted from the C-terminal region of Ndel1 (Ndel1^ΔCTCC^) (Fig. 1*B*) (38, 43, 44, 50). All Ndel1 constructs have HaloTags at their N termini and 6X-His tags at their C termini (51).

First, we determined the binding affinities between each Ndel1 construct and dynein to determine the relative contribution of each of the dynein-binding sites of Ndel1. To do this, we conjugated increasing amounts of Ndel1 to magnetic beads *via* the HaloTag and quantified dynein depletion *via* SDS-PAGE. We found that FL-Ndel1 bound dynein with a *K*_*d*_ of ∼33 nM (Figs. 1*C* and S1*E*). Both NT-Ndel1 and Ndel1^ΔCTCC^ displayed a reduced affinity for dynein (*K*_*d*_ of 79 nM and 55 nM, respectively) (Figs. 1*C* and S1, *F* and *G*). Together, these results suggest that regions in the N-terminal and C-terminal halves of Ndel1 both contribute to interaction with dynein. FL-Ndel1^E48A^ did not appreciably bind dynein (Fig. S1*H*), which indicates that the interaction between the C-terminal half of Ndel1 and dynein is not sufficiently strong to promote binding in the absence of a productive interaction between Ndel1 and the intermediate chain.

Previous work suggested that in the absence of any additional factors, mammalian Ndel1 and Nde1 reduce dynein’s affinity for microtubules (37, 46, 52, 53). Using single-molecule total internal reflection fluorescence microscopy (smTIRF), we monitored the microtubule-binding activity of tetramethylrhodamine (TMR)-labeled dynein in the absence and presence of FL-Ndel1. We observed that FL-Ndel1 did not alter the density of single molecules of dynein on microtubules, indicating that Ndel1 does not modulate dynein’s microtubule-binding affinity (Fig. S1*I*). This finding is consistent with what has been observed with yeast proteins (36).

Next, we investigated if Ndel1 affects the motility of activated dynein complexes. To do this, we assembled TMR- or Alexa647-labeled dynein, purified dynactin, and the purified recombinant adaptor, BicD2, into complexes (DDB) (Fig. S1*D*). Because many FL adaptors display autoinhibition, we used a well-characterized truncation of BicD2 containing amino acids 25 to 398 (hereafter called BicD2) (16, 54, 55, 56). We next incubated DDB with FL-Ndel1 or FL-Ndel1^E48A^ for 10 min to allow samples to reach equilibrium and then imaged motility of DDB using smTIRF. We observed that FL-Ndel1, but not FL-Ndel1^E48A^, significantly reduced the velocity, run length, and landing rate of processive motile events compared with DDB alone (Figs. 1, *D*–*F* and S2*A*). We did not observe FL-Ndel1 comigrating with processive events, suggesting that Ndel1 does not alter motility by remaining bound to the moving complex (Fig. S2*B*).

To determine the relative contribution of the C-terminal binding site of Ndel1 to its effect on dynein motility, we repeated the smTIRF motility experiments with NT-Ndel1 and NT-Ndel1^E48A^ (Fig. S2*C*). As expected, NT-Ndel1^E48A^ did not affect any motility parameters (Fig. 1, G–I). Interestingly, NT-Ndel1 had the same effect on velocity as FL-Ndel1, showing an approximately twofold reduction compared with DDB alone (Fig. 1, *D* and *G*). NT-Ndel1 also reduced the landing rate of processive events, but to a lesser extent than FL-Ndel1 (compare an approximately fivefold reduction in landing rate with FL-Ndel1 to an approximately twofold reduction with NT-Ndel1) (Fig. 1, F and I). NT-Ndel1 did not significantly reduce the run length of motile events compared with DDB alone (Fig. 1*H*).

Together, these data suggest that Ndel1 reduces the number of activated dynein complexes as well as the velocity and run length of processive DDB. Because both NT-Ndel1 and FL-Ndel1 reduced velocity and total processive events, we reason that the inhibitory effect of Ndel1 is mediated by its N-terminal intermediate chain–binding site. The increased inhibition observed with FL-Ndel1 compared with NT-Ndel1 is consistent with the *K*_*d*_ measurements that show that the C-terminal half of Ndel1 increases its association with dynein (Fig. 1*C*). Thus, our data suggest that the C-terminal region of Ndel1 increases the potency of inhibition, but that the N-terminal coiled coil of Ndel1 is necessary and sufficient.

### Ndel1 competes with dynactin and adaptor for dynein binding

**Figure 2:**
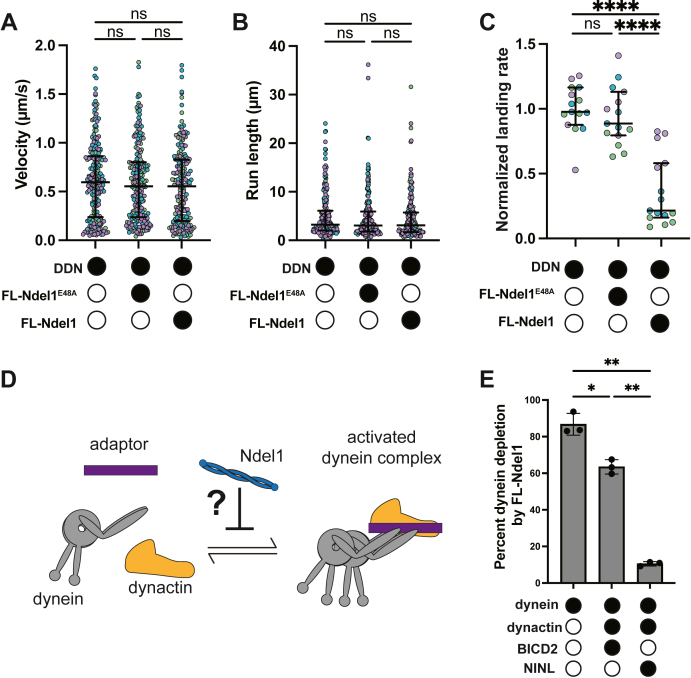
**Ndel1 differentially affects activated complexes formed with different adaptors.***A*, single-molecule velocity of DDN complexes in the absence (*white circles*) or the presence (*black circles*) of 300 nM FL-Ndel1^E48A^ or FL-Ndel1. n = 214 (no Ndel1); 203 (FL-Ndel1^E48A^); 194 (FL-Ndel1). Error bars are median  ±  interquartile range. Statistical analysis was performed using a Kruskal–Wallis with Dunn’s multiple comparisons test. *p* Values: no Ndel1 *versus* FL-Ndel1 = 0.4462; others are >0.9999. *B*, single-molecule run lengths of DDN complexes in the absence (*white circles)* or the presence (*black circles*) of 300 nM FL-Ndel1^E48A^ or FL-Ndel1. n = 214 (no Ndel1); 203 (FL-Ndel1^E48A^); 194 (FL-Ndel1). Error bars are median  ±  interquartile range. Statistical analysis was performed using a Kruskal–Wallis with Dunn’s multiple comparisons test. *p* Values: no Ndel1 *versus* FL-Ndel1 = 0.2995; no Ndel1 *versus* FL-Ndel1^E48A^ >0.9999; FL-Ndel1^E48A^*versus* FL-Ndel1 >0.9999. *C*, single-molecule events per micrometer of microtubule per nanometer dynein for DDN complexes in the absence (*white circles*) or the presence (*black circles*) of 300 nM FL-Ndel1^E48A^ or FL-Ndel1. Data are normalized to the no Ndel1 control for each replicate. n = 15 microtubules per condition. Error bars are median  ±  interquartile range. Statistical analysis was performed using a Brown–Forsythe and Welch ANOVA with Dunnet’s T3 multiple comparisons test. *p* Values: ns = 0.8441; ∗∗∗∗ < 0.0001. *D*, schematic of the assay to test if Ndel1 affects formation of activated dynein complexes. *E*, percentage of dynein bound to FL-Ndel1-conjugated beads in the absence (*white circles*) or the presence (*black circles*) of dynactin, BicD2, and NINL. Statistical analysis was performed using a Brown–Forsythe and Welch ANOVA with Dunnet’s T3 multiple comparisons test. *p* Values: ∗0.0259; ∗∗(dynein *versus* DDN) = 0.0044; ∗∗(DDB *versus* DDN) = 0.0042.

We next asked if Ndel1 had an inhibitory effect on dynein activated with different adaptors. Here, we reconstituted dynein motility with the adaptor ninein-like (NINL) and dynactin (together refered to as DDN) and determined if FL-Ndel1 affected DDN motility (Figs. 2, *A*–*C*, S2*D*, and S1*D*). As with BicD2, we used a truncation of NINL (amino acids 1–702) to avoid autoinhibition (16, 57, 58). As was observed with DDB, FL-Ndel1 reduced the landing rate of processive DDN events (Fig. 2*C*). Unlike with DDB, however, FL-Ndel1 had little effect on the velocity or run length of processive DDN complexes (Fig. 2, *A* and *B*).

We reasoned that the reduction in processive events caused by Ndel1 with both adaptors could be explained if Ndel1 acted upstream of activation and disfavored the formation of the activated dynein complex (Fig. 2*D*). To test this hypothesis, we conjugated FL-Ndel1 *via* the C-terminal 6X-His tag to magnetic beads and monitored its ability to bind and deplete dynein from solution. Here, we incubated FL-Ndel1-beads with dynein alone; dynein, dynactin, and BicD2; or dynein, dynactin, and NINL. FL-Ndel1 depleted over 80% of the dynein in solution in the absence of dynactin and adaptor (Fig. 2*E*). Inclusion of dynactin and BicD2 resulted in less binding between dynein and Ndel1, with a depletion of ∼65% of the dynein (Fig. 2*E*). Remarkably, only ∼11% of the total dynein was depleted by FL-Ndel1 in the presence of dynactin and NINL (Fig. 2*E*). Because dynactin and either adaptor reduced dynein’s binding affinity for FL-Ndel1, these results suggest that Ndel1 competes with dynactin and adaptors for dynein binding. This result supports the model that Ndel1 disfavors complex formation. These results also highlight that DDN is more refractory to Ndel1 inhibition than DDB, which may explain why DDN velocity and run length are less affected by Ndel1 than DDB.

### Phosphomimetic Ndel1 mutations enhance dynein inhibition by Ndel1

**Figure 3:**
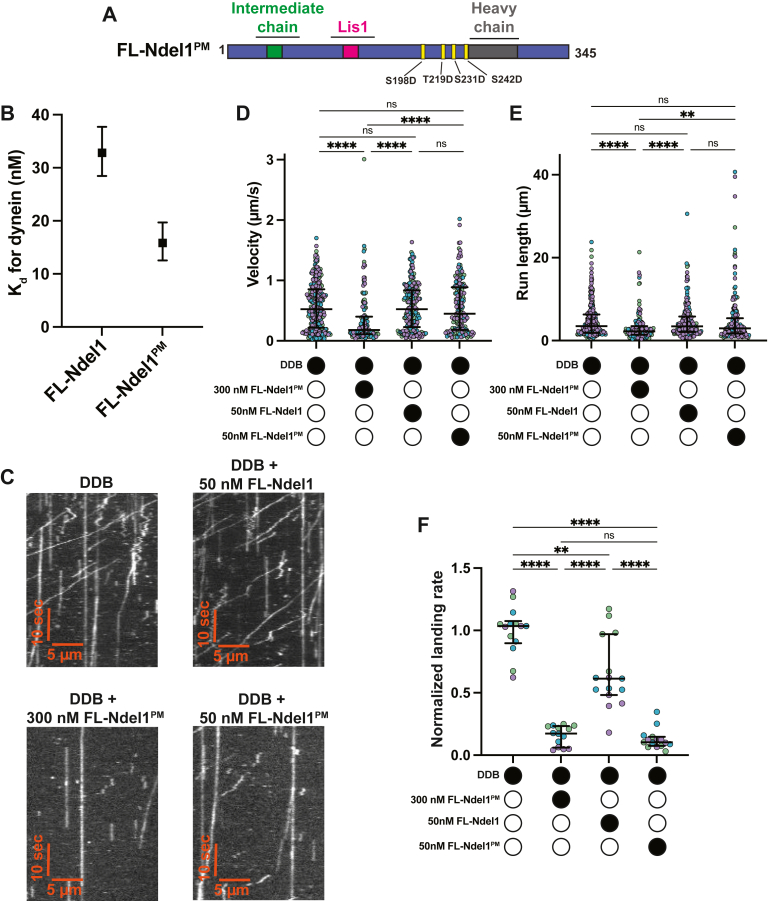
**Phosphomimetic mutations in the C-terminal region of Ndel1 increase the efficacy of activated dynein complex inhibition.***A*, schematic of FL-Ndel1^PM^. *B*, *K*_*d*_s between dynein and FL-Ndel1 constructs. Error bars indicate 95% confidence intervals. FL-Ndel1 data previously shown in Figure 1*C*. *C*, example kymographs of DDB in the presence and absence of FL-Ndel1^PM^ and FL-Ndel1. *D*, single-molecule velocity of DDB complexes in the absence (*white circles*) or the presence (*black circles*) of FL-Ndel1^PM^ or FL-Ndel1 at the concentrations indicated. n = 276 (no Ndel1); 178 (50 nM FL-Ndel1^PM^); 221 (50 nM FL-Ndel1); 138 (300 nM FL-Ndel1^PM^). Error bars are median  ±  interquartile range. Statistical analysis was performed using a Kruskal–Wallis with Dunn’s multiple comparisons test. *p* Values: ns > 0.9999; ∗∗∗∗ < 0.0001. *E*, single-molecule run lengths of DDB complexes in the absence (*white circles*) or the presence (*black circles*) of FL-Ndel1^PM^ or FL-Ndel1 at the concentrations indicated. n = 276 (no Ndel1); 178 (50 nM FL-Ndel1^PM^); 221 (50 nM FL-Ndel1); 138 (300 nM FL-Ndel1^PM^). Error bars are median  ±  interquartile range. Statistical analysis was performed using a Kruskal–Wallis with Dunn’s multiple comparisons test. *p* Values: ns (no Ndel1 *versus* 50 nM FL-Ndel1) >0.9999; ns (no Ndel1 *versus* 50 nM Ndel1^PM^) = 0.3272; ns (50 nM Ndel1 *versus* 50 nM Ndel1^PM^) = 0.6025; ∗∗0.0058; ∗∗∗∗ < 0.0001. *F*, single-molecule events per micrometer of microtubule per nanometer dynein for DDB complexes in the absence (*white circles*) or the presence (*black circles*) of FL-Ndel1^PM^ or FL-Ndel1 at the concentrations indicated. Data are normalized to the no Ndel1 control for each replicate. n = 15 microtubules per condition. Error bars are median  ±  interquartile range. Statistical analysis was performed using a Brown–Forsythe and Welch ANOVA with Dunnet’s T3 multiple comparisons test. *p* Values: ns = 0.8912; ∗∗0.0041; ∗∗∗∗ < 0.0001. DDB, dynein–dynactin–BicD2; FL, full length.

Ndel1 and its paralog Nde1 are phosphoproteins (59). Cell-based studies have revealed that phosphorylation of Ndel1 and Nde1 by many different kinases modulates their subcellular localization or their ability to bind dynein or Lis1 (60, 61, 62, 63, 64). Many kinases target regions in Ndel1 and Nde1 in or around the C-terminal coiled coil that contains the purported heavy chain–binding site (Figs. 3*A* and S3*A*) (60, 64). Phosphorylation of the C-terminal region of Ndel1 regulates the ability of Ndel1 to promote the correct subcellular localization of dynein and/or Lis1. For example, phosphorylation by CDK5 and CDK1 enables the Ndel1-mediated recruitment of dynein or Lis1 to the nuclear pore and kinetochore (60, 61). In addition, in numerous cell-based assays, CDK5 phosphorylation of Ndel1 has been shown to positively regulate association with dynein and dynein-driven cargo trafficking; however, *in vitro* experiments do not support this observation (43, 60, 64, 65).

To explore the effect of phosphorylation on the ability of Ndel1 to modulate dynein motility, we purified a FL-Ndel1 construct with the four sites phosphorylated by CDK1 (amino acids S198, T219, S231, and S242) mutated to aspartic acid to mimic phosphorylation (FL-Ndel1^PM^) (Figs. 3*A* and S1*D*). First, we determined that FL-Ndel1^PM^ binds dynein with a twofold higher affinity than FL-Ndel1 (Figs. 3*B* and S3*B*) using the quantitative binding assay outlined previously. Next, we determined if FL-Ndel1^PM^ functioned like FL-Ndel1 in DDB motility assays (Fig. 3, *C*–*F*). At the concentration of Ndel1 used in previous smTIRF assays (300 nM), FL-Ndel1^PM^ caused a decrease in the run length of processive events that was comparable to what we observed with FL-Ndel1 (Figs. 3, *C* and *E*, 1E, and S2*A*). FL-Ndel1^PM^ reduced the velocity and landing rate to a slightly greater extent than FL-Ndel1 (Figs. 3, *C*, *D*, and *F*, 1, *D* and *F* ,and S2*A*). To probe how much more effective the FL-Ndel1^PM^ construct was, we reduced the concentration of FL-Ndel1^PM^ and FL-Ndel1 to 50 nM and repeated the smTIRF motility experiments with DDB. At the reduced concentration, neither FL-Ndel1^PM^ nor FL-Ndel1 had a statistically significant reduction in velocity or run length (Figs. 3, *C*–*E*, 1, *D* and *E*, and S2*A*). Remarkably, inclusion of 50 nM FL-Ndel1^PM^ reduced the processive landing rate of DDB by 10-fold, whereas 50 nM FL-Ndel1 reduced the landing rate by only 1.7-fold (Fig. 3, *C* and *F*). We conclude that the phosphomimetic mutations in Ndel1 promote its binding to dynein and thereby increase its ability to inhibit the formation of the activated dynein complex.

### Lis1 interactions with Ndel1 and dynein are mutually exclusive

**Figure 4:**
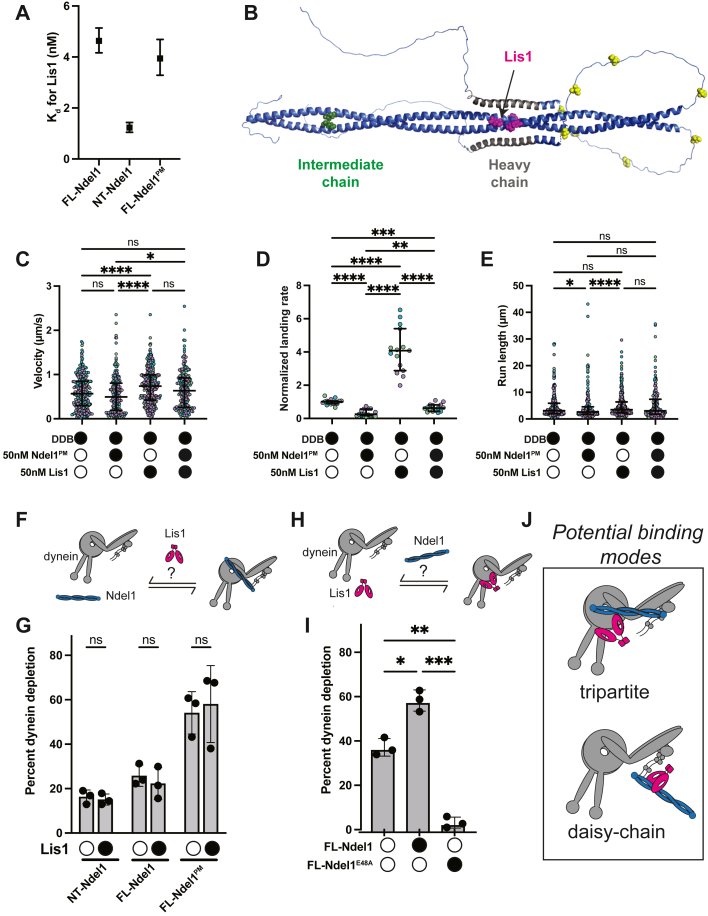
**Ndel1 tethers Lis1 and dynein while preventing Lis1-mediated dynein activation.***A*, *K*_*d*_s between Lis1 and Ndel1 constructs. Error bars indicate 95% confidence intervals. *B*, AlphaFold model with the FL-Ndel1 C terminus folded back. *Green* indicates IC-binding region, *pink* indicates Lis1-binding region, *gray* indicates heavy chain–binding region, and *yellow* indicates CDK1 phosphorylation sites. *C*, single-molecule velocity of DDB complexes in the absence (*white circles*) or the presence (*black circles*) of 50 nM FL-Ndel1^PM^ and Lis1. n = 256 (no Ndel1/Lis1); 200 (FL-Ndel1^PM^); 351 (Lis1); 200 (FL-Ndel1^PM^+Lis1). Error bars are median  ±  interquartile range. Statistical analysis was performed using a Kruskal–Wallis with Dunn’s multiple comparisons test. *p* Values: ns (no Ndel1/Lis1 *versus* FL-Ndel1^PM^) = 0.3759; ns (no Ndel1/Lis1 *versus* FL-Ndel1^PM^ + Lis1) >0.9999; ns (Lis1 *versus* FL-Ndel1^PM^ + Lis1) = 0.0540; ∗0.0172; ∗∗∗∗ < 0.0001. *D*, single-molecule events per micrometer of microtubule per nanometer dynein for DDB complexes in the absence (*white circles*) or the presence (*black circles*) of 50 nM FL-Ndel1^PM^ and Lis1. Data are normalized to the no Ndel1 control for each replicate. n = 15 microtubules per condition. Error bars are median  ± interquartile range. Statistical analysis was performed using a Brown–Forsythe and Welch ANOVA with Dunnet’s T3 multiple comparisons test. *p* Values: ∗∗0.0027; ∗∗∗0.0004; ∗∗∗∗ < 0.0001. *E*, single-molecule run lengths of DDB complexes in the absence (*white circles*) or the presence (*black circles*) of 50 nM FL-Ndel1^PM^ and Lis1. n = 256 (no Ndel1/Lis1); 200 (FL-Ndel1^PM^); 351 (Lis1); 200 (FL-Ndel1^PM^+Lis1). Error bars are median  ±  interquartile range. Statistical analysis was performed using a Kruskal–Wallis with Dunn’s multiple comparisons test. *p* Values: ns (no Ndel1/Lis1 *versus* Lis1) = 0.7162; ns (no Ndel1/Lis1 *versus* FL-Ndel1^PM^+Lis1) >0.9999; ns (FL-Ndel1^PM^*versus* FL-Ndel1^PM^ + Lis1) = 0.1069; ns (Lis1 *versus* FL-Ndel1^PM^ + Lis1) = 0.5455; ∗0.0367; ∗∗∗∗ < 0.0001. *F*, schematic of the assay to test if Lis1 affects the ability of Ndel1 to bind dynein. *G*, percent of dynein bound to Ndel1-conjugated beads in the absence (*white circles*) or the presence (*black circles*) of Lis1. Statistical analysis was performed using a Brown–Forsythe and Welch ANOVA with Dunnet’s T3 multiple comparisons test. *p* Values: NT-Ndel1 = 0.9300; FL-Ndel1 = 0.8589; FL-Ndel1^PM^ = 0.9770. *H*, schematic of the assay to test if Ndel1 affects the ability of Lis1 to bind dynein. *I*, percent of dynein bound to Lis1-conjugated beads in the absence (*white circles*) or the presence (*black circles*) of FL-Ndel1 or FL-Ndel1^E48A^. Statistical analysis was performed using a Brown–Forsythe and Welch ANOVA with Dunnet’s T3 multiple comparisons test. *p* Values: ∗0.0106; ∗∗0.0026; ∗∗∗0.0009. *J*, schematics of the tripartite and daisy-chain dynein-Lis1-Ndel1 complexes. FL, full length.

The ability of Ndel1 to bind to Lis1 is well documented; however, the structural basis for their interaction is poorly defined (25, 37, 39, 44, 66, 67). To dissect the molecular determinants of the interaction between Lis1 and Ndel1, we determined the binding affinity between Lis1 and FL-Ndel1, NT-Ndel1, and FL-Ndel1^PM^. We conjugated each construct of Ndel1 to magnetic resin *via* the N-terminal HaloTag and monitored depletion of Lis1 from solution. We performed the initial binding experiments at 36 mM KCl, which matches the experimental conditions for all previous binding experiments and the smTIRF motility assays. At 36 mM KCl, the *K*_*d*_ between Lis1 and Ndel1 was far too low to accurately measure in the bead-based assay (data not shown). Therefore, we increased the ionic strength of the final assay buffer to 150 mM KCl and repeated the binding experiments. We found that all constructs of Ndel1 bound Lis1 with high affinity. Both FL-Ndel1 and FL-Ndel1^PM^ had a *K*_*d*_ of ∼4 nM for Lis1 (Figs. 4*A* and S4, *A* and *B*). These data suggest that phosphomimetic mutations of Ndel1 at the CDK1 target sites do not affect binding to Lis1. Interestingly, NT-Ndel1 exhibited an increased affinity for Lis1, with a *K*_*d*_ of ∼1.2 nM (Figs. 4*A* and S4*C*), which shows that removing the C terminus of Ndel1 increases its association with Lis1. This result is consistent with crosslinking-mass spectrometry data that suggest that the C-terminus of Ndel1 can fold back toward the N-terminal coiled coil and partially occlude the Lis1-binding site (68). To further validate this model, we used AlphaFold to predict the structure of FL-Ndel1 (69, 70, 71). We found that the C-terminal half of Ndel1 (that contains the predicted heavy chain–binding site) folds back and interacts with the mapped Lis1-binding site (Figs. 4*B* and S4*D*). AlphaFold predictions of just the C-terminal half of Ndel1 (in the absence of the N-terminal portion) indicate that the putative heavy chain–binding site assumes a coiled-coil fold (Fig. S1, *B* and *C*). Given the two conformations of the C-terminal region of Ndel1 predicted by AlphaFold, it is possible that Ndel1 exhibits a conformational equilibrium that regulates binding to Lis1 (Fig. S4*E*).

*Saccharomyces cerevisiae* Ndel1 and Lis1 (Ndl1 and Pac1 in yeast, respectively) have a synergistic effect on Pac1-mediated alteration of dynein velocity (36). Because yeast dynein exhibits processive motility on its own, previous experiments that tested the effect of Ndl1 and Pac1 on dynein were conducted in the absence of dynactin and an adaptor (36, 72). Inclusion of Pac1 in this context reduces the velocity of dynein, and Ndl1 amplifies this effect (16, 36, 37). To test if mammalian Lis1 and Ndel1 showed synergistic activity, we performed the smTIRF motility assay with DDB, 50 nM FL-Ndel1^PM^, and/or 50 nM Lis1 (Figs. 4, *C*–*E* and S4*F*). As previously observed, Lis1 alone resulted in DDB complexes that moved with a faster velocity and exhibited a higher landing rate (Fig. 4, *C* and *D*) (16, 17, 21, 22). At this concentration, Lis1 did not significantly increase the run length of processive events (Fig. 4*E*). Next, we performed the DDB smTIRF motility experiments with equimolar FL-Ndel1^PM^ and Lis1. If Ndel1 tethers Lis1 to dynein as has been proposed, we would anticipate that Lis1 and FL-Ndel1^PM^ should activate dynein motility (*i.e.*, increase velocity, landing rate, and/or run length) to a greater extent than what was observed with Lis1 alone. Instead, we observed that the negative FL-Ndel1^PM^ effect was attenuated by the positive Lis1 effect, with resulting events moving at velocities that were not significantly different than DDB alone (Fig. 4*C*). Remarkably, we observed that the inhibitory effect of FL-Ndel1^PM^ outweighed the activating effect of Lis1 with respect to landing rate. Compared with DDB alone, Lis1 resulted in an approximately 4-fold increase in landing rate, whereas FL-Ndel1^PM^ and Lis1 together exhibited approximately 2-fold reduction in landing rate (Fig. 4*D*). These results suggest that Ndel1 and Lis1 do not interact synergistically to increase dynein activation and that the Ndel1-mediated inhibition of DDB assembly prevents Lis1 from promoting complex formation.

We have observed that Lis1 binds Ndel1 directly, and that the N and C termini of Ndel1 both contribute to dynein binding (Fig. 1*C*). It is also well established that Lis1 binds dynein’s motor domain at the interface of AAA3 and AAA4 (AAA3/4) and along the stalk that emanates from AAA4 and connects dynein’s ATPase ring to the microtubule-binding domain (Fig. S1*A*) (16, 18, 20). We set out to dissect if Ndel1, Lis1, and dynein mutually influence each other’s binding. First, we asked if Lis1 affects Ndel1–dynein interaction (Fig. 4, *F* and *G*). We conjugated NT-Ndel1, FL-Ndel1, or FL-Ndel1^PM^ to magnetic beads and monitored the amount of dynein depleted by each Ndel1 construct in the absence and presence of Lis1. Each Ndel1 construct depleted different amounts of dynein from solution, which is consistent with the measured *K*_*d*_ values (Figs. 1*C* and 3B). However, we observed that Lis1 had no effect on the ability of any of the Ndel1 constructs to deplete dynein (Fig. 4*G*). This suggests that Lis1 does not regulate the Ndel1–dynein interaction.

Next, we asked if Ndel1 influences the Lis1–dynein interaction (Fig. 4, H and I). We conjugated Lis1 to magnetic beads *via* an N-terminal HaloTag. We incubated dynein alone or with tagless FL-Ndel1 (*i.e.*, no HaloTag) and asked if Ndel1 affects the amount of dynein depleted by Lis1. In these experiments, in the absence of FL-Ndel1, we observed ∼40% of the dynein was depleted by Lis1, which is consistent with the Lis1–dynein *K*_*d*_ measured (Figs. 4*I* and S4*G*). Inclusion of FL-Ndel1 increased the amount of dynein depleted by Lis1 to ∼60% (Fig. 4*I*). This suggests that Ndel1 increases the association of dynein and Lis1, which is consistent with previous reports (36, 37).

There are two possible explanations for the apparent increased affinity between Lis1 and dynein in the presence of FL-Ndel1. First, Ndel1, dynein, and Lis1 could assemble in a tripartite structure, with Lis1 bound to dynein at the AAA3/4 and stalk sites, whereas Ndel1 bridges the dynein motor to interact with the intermediate chain and Lis1 simultaneously (Fig. 4*J*, *top*). Second, it is possible that Lis1 cannot bind dynein and Ndel1 at the same time and, instead, Ndel1 “daisy chains” Lis1 to dynein (Fig. 4*J*, *bottom*). This would lead to an increased apparent affinity between Lis1–dynein because Ndel1 binds dynein with a higher affinity than Lis1 does (compare a *K*_*d*_ of 33 nM for dynein–Ndel1 to 101 nM for dynein–Lis1), and Ndel1 and Lis1 are always bound in these experiments given their high-affinity interaction (Figs. 1*C*, S4*G*, and 4A). Here, Ndel1 would be bound to Lis1 and dynein simultaneously without Lis1 engaging the motor at the AAA3/4 and stalk sites (Fig. 4*J*, *bottom*).

To differentiate between the two possible binding modes, we employed FL-Ndel1^E48A^, which disrupts binding to dynein intermediate chain. If the dynein–Lis1–Ndel1 complex is tripartite, FL-Ndel1^E48A^ would reduce the Lis1–dynein binding to the levels observed when no Ndel1 was included (∼40%). However, if Lis1 cannot bind dynein and Ndel1 at the same time and the increased affinity is due to an Ndel1 daisy chain, then FL-Ndel1^E48A^ would inhibit Lis1–dynein binding. When we performed the experiment with FL-Ndel1^E48A^, we observed almost no dynein depletion by Lis1 (Fig. 4*I*). This result supports the daisy-chain model (Fig. 4*J*, *bottom*) and suggests that Lis1 cannot bind Ndel1 and dynein at the same time. This finding is consistent with a recent study that also showed that Lis1 does not bind Ndel1 and dynein concurrently (48). The daisy-chain model of Lis1–dynein–Ndel1 binding also explains the dominant effect of FL-Ndel1^PM^ over Lis1 in the landing rate of DDB measured in the smTIRF motility experiments. The Lis1 residues that contribute to an interaction with dynein’s stalk are very close to residues that mediate interaction with the Ndel1 paralog, Nde1 (Fig. S4*H*) (20, 66, 67), suggesting an overlapping binding site. In addition, in a recent study, Okada *et al.* (48) observed that mutations in Lis1 that disrupt binding to AAA3/4 abrogate Ndel1–Lis1 association, which also supports the hypothesis that Lis1 uses a close site or an overlapping site to bind Ndel1 and dynein.

## Discussion

We set out to explore how the core dynein regulator, Ndel1, affects activation of dynein motility. We found that the C-terminal half of Ndel1 functions in a regulatory capacity to modulate binding to both dynein and Lis1. First, we confirmed that the C-terminal coiled coil of Ndel1 participates in dynein binding (Fig. 1*C*) and found that phosphomimetic mutations in the C-terminus that mimic CDK1 phosphorylation positively regulate binding to dynein (Fig. 3*B*). Because many kinases target similar residues as CDK1, we propose that phosphorylation by multiple kinases may promote increased dynein–Ndel1 interaction (Fig. S3*A*). Further work is needed to understand how the C-terminal coiled-coil of Ndel1 promotes dynein binding and to determine the structural effect of phosphorylation. Our finding that the C-terminal half of Ndel1 negatively regulates association with Lis1 (Fig. 4*A*) suggests that FL-Ndel1 is autoinhibited with respect to Lis1 binding. Structural predictions and previous studies suggest that the C-terminus of Ndel1 contains a stretch of amino acids that can exist as a coiled coil or fold back and interact with the interface that Ndel1 uses to bind Lis1 (Figs. S1, *B* and *C*, Fig. 4*B*, and S4*D*) (26, 68). We posit that Ndel1 may exploit different conformational states to regulate association with Lis1 (Fig. S4*E*). More studies will be required to test this hypothesis.

Our work also shows that Ndel1 directly disfavors the formation of the activated dynein complex and that the N-terminal coiled coil is necessary and sufficient to mediate this effect (Fig. 1, D–I). Previous reports have shown that Ndel1 and the p150 subunit of dynactin compete for binding to the N terminus of the intermediate chain because they have overlapping and yet nonidentical binding sites (45, 47). Although the p150–intermediate chain interface is not resolved in any published structures of dynein–dynactin–adaptor, a recent study shows that the interaction between p150 and the intermediate chain promotes or stabilizes the activated dynein complex (48). Therefore, we suggest that Ndel1 disfavors complex formation by competing with p150 for intermediate chain binding. We found that the C-terminal coiled-coil of Ndel1 promotes binding to dynein, and phosphomimetic mutations in the C-terminus can amplify the inhibitory effect (Figs. 1, *C*–*F* and 3, *B*–*F*). Therefore, we suggest that the C-terminal half of Ndel1 acts to stabilize the interaction with dynein and provide a mechanism for the magnitude of inhibition to be modulated by phosphorylation. Exploring the role of the p150–intermediate chain interaction in activated complex formation will be critical to understand how Ndel1 fits into the current model of dynein activity. We also observe that Ndel1 inhibits the formation of activated dynein complexes to different extents, depending on the identity of the adaptor (Fig. 2). This is likely because different adaptors form activated complexes with varying stabilities. This result suggests that Ndel1–dynein interaction in cells may have a different outcome depending on the adaptor present.

In addition, our work shows that Ndel1 also inhibits dynein activation by preventing the direct association of dynein and Lis1 (Fig. 4*I*). We observe that though Ndel1 can bind dynein and Lis1 at the same time, Lis1 does not bind both Ndel1 and dynein concurrently. This competition is likely because dynein and Ndel1 have close or overlapping binding sites on Lis1. Structural studies will be required to fully test this theory.

**Figure 5:**
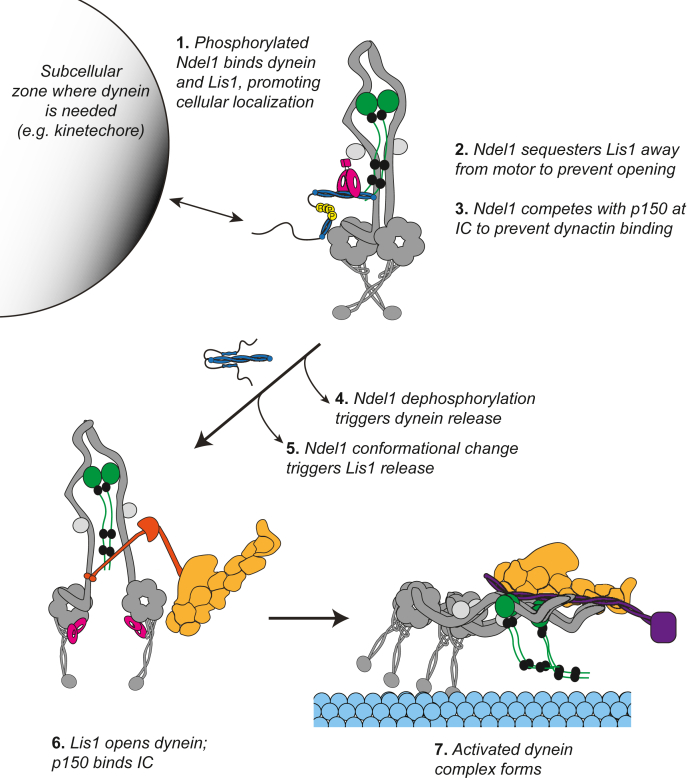
**Model of how Ndel1 regulates the formation of activated dynein complexes through its interaction with dynein and Lis1.** Step 1: Ndel1 and its paralog Nde1 stabilize dynein and Lis1 at multiple cellular regions, including the kinetochore and nuclear pore (5, 28, 60). Phosphorylation by CDK5 and/or CDK1 (which target nearly identical amino acids in Ndel1) are critical for Ndel1-mediated recruitment of dynein and Lis1 (60, 61). Steps 2 and 3: Ndel1 simultaneously binds Lis1 and sequesters it from its AAA3/4 and stalk binding sites on dynein and inhibits the interaction between p150 and the intermediate chain, thus holding dynein in an inactive state. Steps 4 and 5: Ndel1 dissociation from Lis1 and dynein is required for dynein activation. This may be controlled by dephosphorylation and a conformational change, which reduces affinity for dynein and Lis1, respectively. How dephosphorylation or conformational change would be triggered is currently unknown. Steps 6 and 7: Dynein activation can proceed as has been described (9, 12, 13, 18, 19, 20, 24, 81). Our data suggest that p150–intermediate chain interaction is critical for formation of the activated dynein complex.

Together, our data show that Ndel1 operates in two distinct ways to disfavor dynein activation: directly by preventing association of the dynein–dynactin–adaptor complex and indirectly by inhibiting the activator Lis1. However, most published cell-based studies suggest that Ndel1 works with Lis1 to positively regulate dynein activation. How can we reconcile our findings with what has been observed in cells? Given that Ndel1 also promotes dynein localization to key subcellular locations, we propose that Ndel1 acts as a scaffold for dynein and Lis1 (Fig. 5, step 1). In this role, we hypothesize that Ndel1 drives dynein–Lis1 colocalization, as has been observed, while simultaneously holding dynein in an inactive state by preventing dynactin binding and Lis1 activity (Fig. 5, steps 1–3) (73). Because the FL-Ndel1^PM^ was a more potent inhibitor of activated dynein complex assembly, we propose that phosphorylation of the C-terminal domain of Ndel1 also regulates its ability to recruit and inhibit dynein. In this model, Ndel1 must unbind from dynein and Lis1 for dynein activation to occur (Fig. 5, steps 4 and 5). We speculate that dephosphorylation of Ndel1 may aid in dissociation from dynein given the increased affinity of the FL-Ndel1^PM^–dynein interaction; however, it is possible that there are other mechanisms to cause Ndel1 release. For example, at the kinetochore, Ndel1 may interact with CENP-F in a manner that attenuates dynein’s ability to traffic checkpoint proteins (28, 29). It is possible that CENP-F–Ndel1 dissociation may promote Ndel1–dynein dissociation and thus dynein activation. We also propose that conformational changes in Ndel1 where the C-terminal tail folds back may facilitate Lis1 release (Fig. 5, step 5). How this conformational change would be triggered is currently unknown. Once uncoupled from Ndel1, dynein activation can proceed (Fig. 5, steps 6 and 7). It is possible that Ndel1 may also help “reset” dynein to an inactive state by destabilizing the activated dynein complex.

Why would a scaffold that holds dynein in an inhibited state ultimately support dynein activation? It is possible that Ndel1-bound dynein and Lis1 are “primed” for activation, forming a structure that is more amenable to activated dynein complex formation than without Ndel1. If this is true, then Ndel1 holds Lis1 and dynein in such a way to increase the effectiveness of Lis1-mediated activated dynein complex formation. It is also possible that Ndel1 promotes activation of dynein by simply increasing the colocalization and thus effective concentrations of dynein and Lis1. Both possibilities are supported by the numerous observations that the requirement for Nde1 or Ndel1 is less stringent when the concentration of Lis1 is elevated (40, 43, 44, 73, 74).

It is intriguing that Ndel1 holds dynein in an inactive state and promotes its localization. Being able to recruit populations of inhibited dynein to cellular structures would enable a concerted activation of all dyneins in a given cellular region. This would be particularly important for events where dynein activation must be precisely timed, like during mitosis. For example, to support the metaphase–anaphase transition, dynein traffics spindle assembly checkpoint proteins away from the kinetochore. Here, both premature and delayed activation of dynein would be disastrous, leading to chromosome missegregation (75). In addition, dynein activation needs to be synchronized across all kinetochores of the cell to facilitate proper chromosome segregation (76). We speculate that Ndel1 release of dynein can function as a timed trigger to promote dynein activation en masse. More work is required to test this model and establish the cellular signals that modulate Ndel1–dynein association.

### Constructs

**Table:** 

Construct	Source
His-ZZ-TEV-SNAPf-DHC1_IC2C_LIC2_Tctex1_Robl1_LC8	Gift from Andrew Carter (Addgene plasmid #111903)
His-ZZ-TEV-Halo-BicD2(25-398)	Gift from Sam Reck-Peterson
His-ZZ-TEV-Halo-NINL(1-702)	Gift from Sam Reck-Peterson
GST-Ndel1-His6	This work
GST-Ndel1(E48A)-His6	This work
ZZ-TEV-Halo-Ndel1-His6	This work
ZZ-TEV-Halo-Ndel1(E48A)-His6	This work
ZZ-TEV-Halo-Ndel1(1-192)-His6	This work
ZZ-TEV-Halo-Ndel1(1-192 E48A)-His6	This work
ZZ-TEV-Halo-Ndel1(Δ256-291)-His6	This work
ZZ-TEV-Halo-Ndel1(S198D; T219D; S231D; S242D)-His6	This work
ZZ-TEV-Lis1	Gift from Sam Reck-Peterson
ZZ-TEV-Halo-Lis1	Gift from Sam Reck-Peterson
ZZ-TEV-SNAP-Lis1	Gift from Sam Reck-Peterson
p62-Halo stable expression HEK293 cell line	Gift from Sam Reck-Peterson

## Experimental procedures

### Protein expression and purification

Dynein, Lis1, and Ndel1 constructs were expressed in Sf9 cells as described (10, 77). Briefly, pACEBac1 plasmid containing the human dynein genes, pFastBac plasmid containing FL Lis1, and pKL plasmid containing Ndel1 and tagged Lis1 constructs were transformed into DH10EmBacY chemically competent cells with heat shock at 42 °C for 15 s followed by incubation at 37 °C and shaking at 220 rpm for 6 h in S.O.C. media (Thermo Fisher Scientific). The cells were plated on LB–agar plates containing kanamycin (50 μg/ml), gentamicin (7 μg/ml), tetracycline (10 μg/ml), Bluo-Gal (100 μg/ml), and IPTG (40 μg/ml). Cells that contained the plasmid of interest were identified with blue/white selection after 48 to 72 h. For FL human dynein constructs, white colonies were tested for the presence of all six dynein genes with PCR. Colonies were grown overnight in LB medium containing kanamycin (50 μg/ml), gentamicin (7 μg/ml), and tetracycline (10 μg/ml) at 37 °C and agitation at 220 rpm. Bacmid DNA was extracted from overnight cultures using isopropanol precipitation as described (9). About 1 × 10^6^ Sf9 cells in 2 ml of media in a 6-well dish were transfected with up to 2 μg of fresh bacmid DNA using FuGene HD transfection reagent (Promega) at a ratio of 3:1 (Fugene reagent:DNA) according to the manufacturer’s directions. Cells were incubated at 27 °C for 3 days without agitation in a humid incubator. Next, the supernatant containing the virus (V0) was harvested by centrifugation (1000*g*, 5 min, 4 °C). About 1 ml of the V0 virus was used to transfect 50 ml of Sf9 cells at 1 × 10^6^ cells/ml to generate the next passage of virus (V1). Cells were incubated at 27 °C for 3 days with shaking at 105 rpm. Supernatant containing V1 virus was collected by centrifugation (1000*g*, 5 min, 4 °C). All V1 were protected from light and stored at 4 °C until further use. To express protein, 4 ml of V1 virus were used to transfect 400 ml of Sf9 cells at a density of 1 × 10^6^ cells/ml. Cells were incubated at 27 °C for 3 days with shaking at 105 rpm and collected by centrifugation (3500*g*, 10 min, 4 °C). The pellet was washed with 10 ml of ice-cold PBS and collected again *via* centrifugation before being flash frozen in liquid nitrogen and stored at −80 °C until needed for protein purification.

All steps for protein purification were performed at 4 °C unless indicated otherwise. For dynein preparation, Sf9 cell pellets were thawed on ice and resuspended in 40 ml of dynein-lysis buffer (50 mM Hepes [pH 7.4], 100 mM sodium chloride, 1 mM DTT, 0.1 mM Mg–ATP, 0.5 mM Pefabloc, 10% [v/v] glycerol) supplemented with one cOmplete EDTA-free protease inhibitor cocktail tablet (Roche) per 50 ml. Cells were lysed with a Dounce homogenizer (ten strokes with a loose plunger followed by 15 strokes with a tight plunger). The lysate was clarified by centrifugation (183,960*g*, 88 min, 4 °C) in a Type 70Ti rotor (Beckman). The supernatant was mixed with 2 ml of IgG Sepharose 6 Fast Flow beads (Cytiva) equilibrated in Dynein-lysis buffer and incubated for 3 to 4 h with rotation along the long axis of the tube. The beads were transferred to a glass gravity column, washed with at least 200 ml of dynein-lysis buffer and 300 ml of tobacco etch virus (TEV) buffer (50 mM Tris–HCl [pH 8.0], 250 mM potassium acetate, 2 mM magnesium acetate, 1 mM EGTA, 1 mM DTT, 0.1 mM Mg–ATP, and 10% [v/v] glycerol). For fluorescent labeling of SNAP tag, dynein-bound beads were mixed with 5 μM SNAP-Cell-TMR or SNAP-AlexaFluor-647 (New England Biolabs) for 10 min at room temperature. Unconjugated dye was removed with a 300 ml wash with TEV buffer at 4 °C. The beads were resuspended in 15 ml of TEV buffer supplemented with 0.5 mM Pefabloc and 0.2 mg/ml TEV protease and incubated overnight with rotation along the long axis of the tube. Cleaved proteins in the supernatant were concentrated with a 100 K molecular weight cutoff (MWCO) concentrator (EMD Millipore) to 500 μl and purified *via* size-exclusion chromatography on a TSKgel G4000SWXL column (TOSOH Bioscience) with GF150 buffer (25 mM Hepes [pH 7.4], 150 mM KCl, 1 mM MgCl_2_, 1 mM DTT) supplemented with 0.1 mM Mg–ATP as the mobile phase at 0.75 ml/min. Peak fractions were collected, buffer exchanged into a GF150 buffer supplemented with 0.1 mM Mg–ATP and 10% glycerol, and concentrated to 0.1 to 0.5 mg/ml using a 100 K MWCO concentrator. Small-volume aliquots were flash frozen in liquid nitrogen and stored at −80 °C.

Lysis and clarification steps for the Ndel1 and Lis1 purifications were similar to the dynein purification except Lis1-lysis buffer (30 mM Hepes [pH 7.4], 50 mM potassium acetate, 2 mM magnesium acetate, 1 mM EGTA, 300 mM potassium chloride, 1 mM DTT, 0.5 mM Pefabloc, 10% [v/v] glycerol supplemented with one cOmplete EDTA-free protease inhibitor cocktail tablet per 50 ml) was used in place of dynein-lysis buffer. The clarified supernatant was mixed with 2 ml of IgG Sepharose 6 Fast Flow beads (GE Healthcare Life Sciences) and incubated for 2 to 3 h with rotation along the long axis of the tube. The beads were transferred to a gravity column, washed with at least 20 ml of Lis1-lysis buffer, 200 ml of Lis1-TEV buffer (10 mM Tris–HCl [pH 8.0], 2 mM magnesium acetate, 150 mM potassium acetate, 1 mM EGTA, 1 mM DTT, 10% [v/v] glycerol) supplemented with 100 mM potassium acetate and 0.5 mM Pefabloc, and 100 ml of Lis1-TEV buffer. For fluorescent labeling of Ndel1, the Ndel1-bound beads were mixed with 5 μM Halo-JFX646 (Lavis Lab) for 10 min at room temperature after the lysis buffer wash. TEV protease was added to the beads at a final concentration of 0.2 mg/ml, and the beads were incubated overnight with rotation along the long axis of the tube. Cleaved Lis1 or Ndel1 in the supernatant was collected and concentrated to 500 μl with a 30 K MWCO concentrator. The concentrated protein was then purified *via* size-exclusion chromatography on a Superose 6 Increase 10/300 GL column (Cytiva) with GF150 buffer as the mobile phase at 0.75 ml/min. For Lis1, the GF150 mobile phase was supplemented with 10% glycerol. SNAP-Lis1 was labeled with SNAP-AlexaFluor 647 (Promega) by incubating with 5 μM dye for 10 min at room temperature prior to size-exclusion chromatography. Peak fractions were collected, concentrated to 0.2 to 1 mg/ml with a 30 K MWCO concentrator, frozen in liquid nitrogen, and stored at −80 °C. Ndel1 purifications were supplemented with 10% glycerol before freezing.

Dynactin was purified from human embryonic kidney 293T cell lines stably expressing p62-Halo-3xFLAG as described (57). Briefly, frozen pellets collected from 160 15 cm plates were resuspended in 80 ml of dynactin-lysis buffer (30 mM Hepes [pH 7.4], 50 mM potassium acetate, 2 mM magnesium acetate, 1 mM EGTA, 1 mM DTT, 10% [v/v] glycerol) supplemented with 0.5 mM Mg-ATP, 0.2% Triton X-100, and 1× cOmplete EDTA-free protease inhibitor cocktail tablets) and rotated along the long axis of the tube for at least 15 min. The lysate was clarified *via* centrifugation (66,000*g*, 30 min, 4 °C) in a Type 70 Ti rotor (Beckman). The supernatant was mixed with 1.5 ml of anti-FLAG M2 affinity gel (Sigma–Aldrich) and incubated overnight with rotation along the long axis of the tube. The beads were transferred to a glass gravity column, washed with at least 50 ml of wash buffer (dynactin-lysis buffer supplemented with 0.1 mM Mg–ATP, 0.5 mM Pefabloc, and 0.02% Triton X-100), 100 ml of wash buffer supplemented with 250 mM potassium acetate, and then washed again with 100 ml of wash buffer. About 1 ml of elution buffer (wash buffer with 2 mg/ml of 3xFLAG peptide) was used to elute dynactin, which was then filtered *via* centrifuging through an Ultrafree-MC VV filter (EMD Millipore) in a tabletop centrifuge according to the manufacturer’s instructions. The filtered dynactin was then diluted to 2 ml in buffer A (50 mM Tris–HCl [pH 8.0], 2 mM magnesium acetate, 1 mM EGTA, and 1 mM DTT) and loaded onto a MonoQ 5/50 GL column (Cytiva) at 1 ml/min. The column was prewashed with 10 column volumes (CVs) of buffer A, 10 CVs of buffer B (50 mM Tris–HCl [pH 8.0], 2 mM magnesium acetate, 1 mM EGTA, 1 mM DTT, 1 M potassium acetate) and then equilibrated with 10 CVs of buffer A. A linear gradient was run over 26 CVs from 35 to 100% buffer B. Pure dynactin eluted between 75 and 80% buffer B. Peak fractions were collected, pooled, buffer exchanged into a GF150 buffer supplemented with 10% glycerol, concentrated to 0.02 to 0.1 mg/ml using a 100 K MWCO concentrator, aliquoted into small volumes, and then flash frozen in liquid nitrogen.

BicD2 and NINL containing amino-terminal HaloTags were expressed in BL-21[DE3] cells (New England Biolabs) at an absorbance at 600 nm of 0.4 to 0.6 with 0.1 mM IPTG for 16 h at 18  °C. Frozen cell pellets from a 1.5 l culture were resuspended in 40 ml of activating-adaptor-lysis buffer (30 mM Hepes [pH 7.4], 50 mM potassium acetate, 2 mM magnesium acetate, 1 mM EGTA, 1 mM DTT, and 0.5 mM Pefabloc, 10% [v/v] glycerol) supplemented with 1× cOmplete EDTA-free protease inhibitor cocktail tablets and 1 mg/ml lysozyme. The resuspension was incubated on ice for 30 min and lysed by sonication. The lysate was clarified by centrifuging at 66,000*g* for 30 min in Type 70 Ti rotor. The clarified supernatant was incubated with 2 ml of IgG Sepharose 6 Fast Flow beads (Cytiva) for 2 h on a roller. The beads were transferred into a gravity-flow column, washed with 100 ml of activating-adaptor-lysis buffer supplemented with 150 mM potassium acetate and 50 ml of cleavage buffer (50 mM Tris–HCl [pH 8.0], 150 mM potassium acetate, 2 mM magnesium acetate, 1 mM EGTA, 1 mM DTT, 0.5 mM Pefabloc, and 10% [v/v] glycerol). The beads were then resuspended and incubated in 15 ml of cleavage buffer supplemented with 0.2 mg/ml TEV protease overnight with rotation along the long axis of the tube. The supernatant containing cleaved proteins was concentrated using a 30 kDa MWCO concentrator to 1 ml, filtered by centrifuging with Ultrafree-MC VV filter (EMD Millipore) in a tabletop centrifuge, diluted to 2 ml in buffer A (30 mM Hepes [pH 7.4], 50 mM potassium acetate, 2 mM magnesium acetate, 1 mM EGTA, 10% [v/v] glycerol, and 1 mM DTT) and injected into a MonoQ 5/50 GL column at 1 m/min. The column was prewashed with 10 CVs of buffer A, 10 CVs of buffer B (30 mM Hepes [pH 7.4], 1 M potassium acetate, 2 mM magnesium acetate, 1 mM EGTA, 10% [v/v] glycerol, and 1 mM DTT) and again with 10 CVs of buffer A. To elute, a linear gradient was run over 26 CVs from 0 to 100% buffer B. The peak fractions containing Halo-tagged activating adaptors were collected and concentrated to using a 30 kDa MWCO concentrator to 0.2 ml, diluted to 0.5 ml in GF150 buffer, and further purified using size-exclusion chromatography on a Superose 6 Increase 10/300 GL column (Cytiva) with GF150 buffer at 0.5 ml/min. The peak fractions were collected, buffer-exchanged into a GF150 buffer supplemented with 10% glycerol, concentrated to 0.2 to 1 mg/ml using a 30 kDa MWCO concentrator, and flash frozen in liquid nitrogen.

### Structure prediction

To predict the structure of FL-Ndel1 and the C-terminal half of Ndel1, we used the COSMIC2 web platform to run the AlphaFold2 Colab Notebook on FL-Ndel1 or FL-Ndel1 (amino acids 194–345) (69, 70, 71).

### Protein binding assays

The binding affinity of Ndel1 constructs for dynein and Lis1 was determined by coupling Ndel1 to 25 μl of Magne HaloTag Beads (Promega) in 2 ml Protein Lo Bind Tubes (Eppendorf) using the following protocol. Beads were washed twice with 1 ml of GF150 without ATP supplemented with 10% glycerol and 0.1% NP-40. Ndel1 was diluted in this buffer to 0, 30, 60, 90, 120, 300, and 600 nM. About 25 μl of diluted Ndel1 was added to the beads and gently shaken for 1 h. About 20 μl of supernatant were then analyzed *via* SDS-PAGE to confirm complete depletion of Ndel1. The Ndel1-conjugated beads were washed once with 1 ml GF150 with 10% glycerol and 0.1% NP-40 and once with 1 ml of binding buffer (30 mM Hepes [pH 7.4], 2 mM magnesium acetate, 1 mM EGTA, 10% glycerol, 1 mM DTT, 1 mg/ml casein, 0.1% NP-40, and 1 mM ADP) supplemented with 36 mM KCl (or 150 mM KCl for high salt Lis1 curves). About 5 nM dynein or Lis1 was diluted in binding buffer, which resulted in binding buffer with 36 mM KCl (or 150 mM KCl for high salt Lis1 curves). About 25 μl of the dynein or Lis1 mixture was added to the beads prebound with Ndel1 and gently agitated for 45 min. After incubation, 20 μl of the supernatant was removed, and 6.67 μl of NuPAGE LDS Sample Buffer (4×) and 1.33 μl of beta-mercaptoethanol was added to each. The samples were boiled for 5 min before running on a 4 to 12% NuPAGE Bis–Tris gel. Depletion was determined using densitometry in ImageJ. For dynein–Ndel1 and dynein–Lis1 binding data, curves were fit in Prism 9 (GraphPad Software, Inc) with a nonlinear regression for one site binding with Bmax set to 1. Because the Lis1–Ndel1 interaction is high affinity and the concentration of Lis1 used in the Lis1–Ndel1 binding assays is comparable to the observed *K*_d_, we fit curves for Lis1–Ndel1 binding data with a quadratic binding equation, as described (78).

Experiments measuring binding between dynein, Lis1, and Ndel1 were performed with the same method and buffers as aforementioned. For the experiment with Lis1 on the beads, 50 nM of Halo-Lis1 was conjugated to the beads and incubated with 5 nM dynein with and without 100 nM of WT and E48A Ndel1. For the experiment with Ndel1 on the beads, 30 nM of Halo-Ndel1 was conjugated to beads and incubated with 5 nM dynein in the presence and absence of 30 nM Lis1.

For experiments investigating the competition between Ndel1 and dynactin–activating adaptor, the methods and buffers were the same as aforementioned. About 90 nM Ndel1 was conjugated to 30 μl of NEBExpress Ni–NTA Magnetic Beads (NEB) and incubated with 5 nM dynein. The appropriate samples were further supplemented with 10 nM dynactin and 150 nM of either BicD2 or NINL. Three or more replicates were performed for all binding assays.

### smTIRF microscopy

Single-molecule imaging was performed with an inverted microscope (Nikon; Ti2-E Eclipse) with a 100× 1.49 numerical aperture oil immersion objective (Nikon, Apo). The microscope was equipped with a LUNF-XL laser launch (Nikon), with 405, 488, 561, and 640 nm laser lines. The excitation path was filtered using an appropriate quad bandpass filter cube (Chroma). The emission path was filtered through appropriate emission filters (Chroma) located in a high-speed filter wheel (Finger Lakes Instrumentation). Emitted signals were detected on an electron-multiplying CCD camera (Andor Technology; iXon Ultra 897). Image acquisition was controlled by NIS Elements Advanced Research software (Nikon).

Single-molecule motility and microtubule-binding assays were performed in flow chambers assembled as described previously (77, 79). Either biotin-PEG-functionalized coverslips (microsurfaces or generated in laboratory) or no. 1 to 1/2 coverslips (Corning) sonicated in 100% ethanol for 10 min were used for the flow-chamber assembly. Taxol-stabilized microtubules with ∼10% biotin–tubulin and ∼10% Alexa488-labeled fluorescent tubulin were prepared as described (36). Flow chambers were assembled with taxol-stabilized microtubules by incubating sequentially with the following solutions, interspersed with two washes with assay buffer (30 mM Hepes [pH 7.4], 2 mM magnesium acetate, 1 mM EGTA, 10% glycerol, and 1 mM DTT) supplemented with 20 μM taxol in between: (1) 1 mg/ml biotin–bovine serum albumin in assay buffer (3 min incubation); (2) 1 mg/ml streptavidin in assay buffer (3 min incubation); and (3) a fresh dilution of taxol-stabilized microtubules in assay buffer (3 min incubation). After flowing in microtubules, the flow chamber was washed twice with assay buffer supplemented with 1 mg/ml casein and 20 μM taxol.

To assemble dynein–dynactin–activating adaptor complexes, purified dynein (10–20 nM concentration), dynactin, and the activating adaptor were mixed at 1:2:10 M ratio and incubated on ice for 10 min. These dynein–dynactin–activating adaptor complexes were then incubated with Ndel1 and/or Lis1 or modified TEV buffer (to buffer match for experiments without Ndel1 or Lis1) for 10 min on ice. Dynactin and the activating adaptors were omitted for the experiments with dynein alone. The mixtures of dynein, dynactin, activating adaptor or dynein alone, and Nde1l/Lis1 were then flowed into the flow chamber assembled with taxol-stabilized microtubules. The final imaging buffer contained the assay buffer supplemented with 20 μM taxol, 1 mg/ml casein, 71.5 mM β-mercaptoethanol, 0.05 mg/ml glucose catalase, 1.2 mg/ml glucose oxidase, 0.4% glucose, and 2.5 mM Mg–ATP. The final concentration of dynein in the flow chamber was 0.5 to 1 nM for experiments with dynein–dynactin–activating adaptor complexes and 0.05 nM for dynein-alone experiments. For standard motility experiments, our final imaging buffer contained 37.5 mM KCl.

For single-molecule motility assays, microtubules were imaged first by taking a single-frame snapshot. Dynein labeled with fluorophores (TMR or Alexa647) was imaged every 300 ms for 3 min. At the end, microtubules were imaged again by taking a snapshot to assess stage drift. Movies showing significant drift were not analyzed. Each sample was imaged no longer than 10 min. For single-molecule microtubule binding assays, the final imaging mixture containing dynein was incubated for an additional 5 min in the flow chamber at room temperature before imaging. After 5 min incubation, microtubules were imaged first by taking a single-frame snapshot. Dynein labeled with fluorophores (TMR or Alexa64) were imaged by taking a single-frame snapshot. Each sample was imaged at two different fields of view, and there were between 15 and 25 microtubules in each field of view.

Kymographs were generated from motility movies, and dynein velocity and run length were calculated from kymographs using custom ImageJ macros as described (80). Only runs longer than eight pixels were included in the analysis. A single pixel size is equal to 157 nm in the *x*-direction and 300 ms in the *y*-direction. Bright protein aggregates, which were defined as molecules 4× brighter than the background, were excluded. Velocity and run length were reported for individual dynein complexes. For the analysis of landing rate, the number of processive events measured on individual microtubules was divided by the length of the microtubule, the concentration of dynein, and the total imaging time. Landing rate was calculated for five microtubules per field of view for each sample. Data were then normalized to the landing rate of the positive control (dynein–dynactin–adaptor complex without Ndel1 and/or Lis1) collected on the same slide. Data plotting and statistical analyses were performed in Prism 9. Three technical replicates, including at least two individual dynein preparations (representing a biological replicate), were performed for each smTIRF motility experiment. Data from individual replicates are shown in different colors *(blue*, *green*, and *purple*) in Figs. 1, D–I, 2, *A*–*C*, 3 *D*–*F*, and 4 *C*–*E*.

## Data availability

All data from this publication can be found in the supporting information data file.

## Supporting information

This article contains supporting information.

## Conflict of interest

The authors declare that they have no conflicts of interest with the contents of this article.

## Supporting information

Supporting Figure S1 *A*, Model of Lis1 favoring open dynein and leading to activated dynein complex formation (16, 17, 19, 23, 24). *B*, Model of the C terminus of Ndel1 (from amino acids 194–345) generated with AlphaFold showing alpha helical structure. Amino acids located at the beginning and end of the coiled-coil are indicated. Amino acids that have been shown to contribute to heavy chain binding are indicated in grey. *C*, Predicted alignment error plot for the AlphaFold model in (*B*). *D*, SDS-PAGE gels of all purified proteins used in this study. *E*, Binding curve between dynein and FL-Ndel1. n = 6. Error bars are mean ± SD. *F*, Binding curve between dynein and NT-Ndel1. n = 4. Error bars are mean ± SD. *G*, Binding curve between dynein and FL-Ndel1^ΔCTCC^. n = 3. Error bars are mean ± SD. *H*, Binding curve between dynein and FL-Ndel1^E48A^. n = 3. Error bars are mean ± SD. 5 nM dynein was used in each binding experiment. *I*, Normalized dynein spots/μm in the absence (*white circles*) and presence (*black circles*) of 300 nM FL-Ndel1. Each point represents a field of view with 15 to 25 microtubules. n = 8. Error bars are mean ± SEM. Statistical analysis was performed using Welch’s T test. *p* values: ns = 0.2641.

Supporting Figure S2 *A*, Example kymographs of DDB with and without 300 nM FL-Ndel1^E48A^ and FL-Ndel1. *B*, Example kymographs of DDB (TMR dynein; *red* in merge) with 10 nM Janelia Fluor-647 labelled FL-Ndel1 (*cyan* in merge). *C*, Example kymographs of DDB with and without 300 nM NT-Ndel1^E48A^ and NT-Ndel1. *D*, Example kymographs of DDN with and without 300 nM FL-Ndel1^E48A^ and FL-Ndel1.

Supporting Figure S3 *A*, Schematics of FL-Ndel1 showing the residues phosphorylated by CDK5, CDK1, Erk2 and Aurora-A. *B*, Binding curve between dynein and FL-Ndel1^PM^. n = 3. Error bars are mean ± SD.

Supporting Figure S4 *A*, Binding curve between Lis1 and FL-Ndel1. n = 6. Error bars are mean ± SD. *B*, Binding curve between Lis1 and FL-Ndel1^PM^. n = 4. Error bars are mean ± SD. *C*, Binding curve between Lis1 and NT-Ndel1. n = 3. Error bars are mean ± SD. *D*, Predicted alignment error plot for the AlphaFold model in Figure 4*B*. *E*, Conformational equilibrium of FL-Ndel1. *F*, Example kymographs of DDB with and without 50 nM FL-Ndel1^PM^ and Lis1. *G*, Binding curve between dynein and Lis1. n = 3. Error bars are mean ± SD. *H*, Model of the Lis1 beta propeller (*pink*) (PDB: 1VYH) showing residues known to interact with Ndel1(*blue*) and dynein’s stalk (*gray*) (20, 66, 67).

## References

[1] Reck-Peterson S.L., Redwine W.B., Vale R.D., Carter A.P. (2018). The cytoplasmic dynein transport machinery and its many cargoes. Nat. Rev. Mol. Cell Biol..

[2] Raaijmakers J.A., Medema R.H. (2014). Function and regulation of dynein in mitotic chromosome segregation. Chromosoma.

[3] Lam C., Vergnolle M.A.S., Thorpe L., Woodman P.G., Allan V.J. (2010). Functional interplay between LIS1, NDE1 and NDEL1 in dynein-dependent organelle positioning. J. Cell Sci..

[4] Robinson J.T., Wojcik E.J., Sanders M.A., McGrail M., Hays T.S. (1999). Cytoplasmic dynein is required for the nuclear attachment and migration of centrosomes during mitosis in Drosophila. J. Cell Biol..

[5] Vergnolle M.A.S., Taylor S.S. (2007). Cenp-F links kinetochores to Ndel1/Nde1/Lis1/dynein microtubule motor complexes. Curr. Biol..

[6] Lipka J., Kuijpers M., Jaworski J., Hoogenraad C.C. (2013). Mutations in cytoplasmic dynein and its regulators cause malformations of cortical development and neurodegenerative diseases. Biochem. Soc. Trans..

[7] Carter A.P. (2013). Crystal clear insights into how the dynein motor moves. J. Cell Sci..

[8] Canty J.T., Tan R., Kusakci E., Fernandes J., Yildiz A. (2021). Structure and mechanics of dynein motors. Annu. Rev. Biophys..

[9] Zhang K., Foster H.E., Rondelet A., Lacey S.E., Bahi-Buisson N., Bird A.W. (2017). Cryo-EM reveals how human cytoplasmic dynein is auto-inhibited and activated. Cell.

[10] Schlager M.A., Hoang H.T., Urnavicius L., Bullock S.L., Carter A.P. (2014). *In vitro* reconstitution of a highly processive recombinant human dynein complex. EMBO J..

[11] McKenney R.J., Huynh W., Tanenbaum M.E., Bhabha G., Vale R.D. (2014). Activation of cytoplasmic dynein motility by dynactin-cargo adapter complexes. Science.

[12] Chaaban S., Carter A.P. (2022). Structure of dynein-dynactin on microtubules shows tandem adaptor binding. Nature.

[13] Urnavicius L., Lau C.K., Elshenawy M.M., Morales-Rios E., Motz C., Yildiz A. (2018). Cryo-EM shows how dynactin recruits two dyneins for faster movement. Nature.

[14] Urnavicius L., Zhang K., Diamant A.G., Motz C., Schlager M.A., Yu M. (2015). The structure of the dynactin complex and its interaction with dynein. Science.

[15] Grotjahn D.A., Chowdhury S., Xu Y., McKenney R.J., Schroer T.A., Lander G.C. (2018). Cryo-electron tomography reveals that dynactin recruits a team of dyneins for processive motility. Nat. Struct. Mol. Biol. U. S. A..

[16] Htet Z.M., Gillies J.P., Baker R.W., Leschziner A.E., DeSantis M.E., Reck-Peterson S.L. (2020). LIS1 promotes the formation of activated cytoplasmic dynein-1 complexes. Nat. Cell Biol..

[17] Elshenawy M.M., Kusakci E., Volz S., Baumbach J., Bullock S.L., Yildiz A. (2020). Lis1 activates dynein motility by modulating its pairing with dynactin. Nat. Cell Biol..

[18] Gillies J.P., Reimer J.M., Karasmanis E.P., Lahiri I., Htet Z.M., Leschziner A.E. (2022). Structural basis for cytoplasmic dynein-1 regulation by Lis1. Elife.

[19] Karasmanis E.P., Reimer J.M., Kendrick A.A., Rodriguez J.A., Truong J.B., Lahiri I. (2022). Lis1 relieves cytoplasmic dynein-1 auto-inhibition by acting as a molecular wedge. bioRxiv.

[20] Reimer J.M., DeSantis M.E., Reck-Peterson S.L., Leschziner A.E. (2022). Structures of human cytoplasmic dynein in complex with the lissencephaly 1 protein. bioRxiv.

[21] Baumbach J., Murthy A., McClintock M.A., Dix C.I., Zalyte R., Hoang H.T. (2017). Lissencephaly-1 is a context-dependent regulator of the human dynein complex. Elife.

[22] Gutierrez P.A., Ackermann B.E., Vershinin M., McKenney R.J. (2017). Differential effects of the dynein-regulatory factor Lissencephaly-1 on processive dynein-dynactin motility. J. Biol. Chem..

[23] Qiu R., Zhang J., Xiang X. (2019). LIS1 regulates cargo-adapter-mediated activation of dynein by overcoming its autoinhibition *in vivo*. J. Cell Biol..

[24] Marzo M.G., Griswold J.M., Markus S.M. (2020). Pac1/LIS1 stabilizes an uninhibited conformation of dynein to coordinate its localization and activity. Nat. Cell Biol..

[25] Derewenda U., Tarricone C., Choi W.C., Cooper D.R., Lukasik S., Perrina F. (2007). The structure of the coiled-coil domain of Ndel1 and the basis of its interaction with Lis1, the causal protein of Miller-Dieker lissencephaly. Structure.

[26] Ye F., Kang E., Yu C., Qian X., Jacob F., Yu C. (2017). DISC1 regulates neurogenesis via modulating kinetochore attachment of Ndel1/Nde1 during mitosis. Neuron.

[27] Bolhy S., Bouhlel I., Dultz E., Nayak T., Zuccolo M., Gatti X. (2011). A Nup133-dependent NPC-anchored network tethers centrosomes to the nuclear envelope in prophase. J. Cell Biol..

[28] Liang Y., Yu W., Li Y., Yu L., Zhang Q., Wang F. (2007). Nudel modulates kinetochore association and function of cytoplasmic dynein in M phase. Mol. Biol. Cell.

[29] Auckland P., Roscioli E., Coker H.L.E., McAinsh A.D. (2020). CENP-F stabilizes kinetochore-microtubule attachments and limits dynein stripping of corona cargoes. J. Cell Biol..

[30] Zhang Y., Chen Z., Wang F., Sun H., Zhu X., Ding J. (2021). Nde1 is a Rab9 effector for loading late endosomes to cytoplasmic dynein motor complex. Structure.

[31] Yi J.Y., Ori-McKenney K.M., McKenney R.J., Vershinin M., Gross S.P., Vallee R.B. (2011). High-resolution imaging reveals indirect coordination of opposite motors and a role for LIS1 in high-load axonal transport. J. Cell Biol..

[32] Li Y.Y., Yeh E., Hays T., Bloom K. (1993). Disruption of mitotic spindle orientation in a yeast dynein mutant. Proc. Natl. Acad. Sci. U. S. A..

[33] Plamann M., Minke P.F., Tinsley J.H., Bruno K.S. (1994). Cytoplasmic dynein and actin-related protein Arp1 are required for normal nuclear distribution in filamentous fungi. J. Cell Biol..

[34] Xiang X., Beckwith S.M., Morris N.R. (1994). Cytoplasmic dynein is involved in nuclear migration in Aspergillus nidulans. Proc. Natl. Acad. Sci. U. S. A..

[35] Xiang X., Osmani A.H., Osmani S.A., Xin M., Morris N.R. (1995). NudF, a nuclear migration gene in Aspergillus nidulans, is similar to the human LIS-1 gene required for neuronal migration. Mol. Biol. Cell.

[36] Huang J., Roberts A.J., Leschziner A.E., Reck-Peterson S.L. (2012). Lis1 acts as a “Clutch” between the ATPase and microtubule-binding domains of the dynein motor. Cell.

[37] McKenney R.J., Vershinin M., Kunwar A., Vallee R.B., Gross S.P. (2010). LIS1 and NudE induce a persistent dynein force-producing state. Cell.

[38] Wang S., Ketcham S.A., Schön A., Goodman B., Wang Y., Yates J. (2013). Nudel/NudE and Lis1 promote dynein and dynactin interaction in the context of spindle morphogenesis. Mol. Biol. Cell.

[39] Efimov V.P., Morris N.R. (2000). The Lis1-related Nudf protein of *Aspergillus nidulans* interacts with the coiled-coil domain of the Nude/Ro11 protein. J. Cell Biol..

[40] Efimov V.P. (2003). Roles of NUDE and NUDF proteins of Aspergillus nidulans: insights from intracellular localization and overexpression effects. Mol. Biol. Cell.

[41] Arthur A.L., Yang S.Z., Abellaneda A.M., Wildonger J. (2015). Dendrite arborization requires the dynein cofactor NudE. J. Cell Sci..

[42] Moon H.M., Youn Y.H., Pemble H., Yingling J., Wittmann T., Wynshaw-Boris A. (2014). LIS1 controls mitosis and mitotic spindle organization via the LIS1–NDEL1–dynein complex. Hum. Mol. Genet..

[43] Żyłkiewicz E., Kijańska M., Choi W.-C., Derewenda U., Derewenda Z.S., Stukenberg P.T. (2011). The N-terminal coiled-coil of Ndel1 is a regulated scaffold that recruits LIS1 to dynein. J. Cell Biol..

[44] Wang S., Zheng Y. (2011). Identification of a novel dynein binding domain in Nudel essential for spindle pole organization in *Xenopus* egg extract. J. Biol. Chem..

[45] Nyarko A., Song Y., Barbar E. (2012). Intrinsic disorder in dynein intermediate chain modulates its interactions with NudE and dynactin∗. J. Biol. Chem..

[46] McKenney R.J., Weil S.J., Scherer J., Vallee R.B. (2011). Mutually exclusive cytoplasmic dynein regulation by NudE-Lis1 and dynactin. J. Biol. Chem..

[47] Jie J., Löhr F., Barbar E. (2017). Dynein binding of competitive regulators dynactin and NudE involves novel interplay between phosphorylation site and disordered spliced linkers. Structure.

[48] Okada K., Iyer B.R., Lammers L.G., Gutierrez P.A., Li W., Markus S.M. (2023). Conserved roles for the dynein intermediate chain and Ndel1 in assembly and activation of dynein. bioRxiv.

[49] Liang Y., Yu W., Li Y., Yang Z., Yan X., Huang Q. (2004). Nudel functions in membrane traffic mainly through association with Lis1 and cytoplasmic dynein. J. Cell Biol..

[50] Sasaki S., Shionoya A., Ishida M., Gambello M.J., Yingling J., Wynshaw-Boris A. (2000). A LIS1/NUDEL/cytoplasmic dynein heavy chain complex in the developing and adult Nervous system. Neuron.

[51] Los G.V., Encell L.P., McDougall M.G., Hartzell D.D., Karassina N., Zimprich C. (2008). HaloTag: a novel protein labeling technology for cell imaging and protein analysis. ACS Chem. Biol..

[52] Yamada M., Toba S., Yoshida Y., Haratani K., Mori D., Yano Y. (2008). LIS1 and NDEL1 coordinate the plus-end-directed transport of cytoplasmic dynein. EMBO J..

[53] Torisawa T., Nakayama A., Furuta K., Yamada M., Hirotsune S., Toyoshima Y.Y. (2011). Functional dissection of LIS1 and NDEL1 towards understanding the molecular mechanisms of cytoplasmic dynein regulation. J. Biol. Chem..

[54] Huynh W., Vale R.D. (2017). Disease-associated mutations in human BICD2 hyperactivate motility of dynein–dynactin. J. Cell Biol..

[55] Sladewski T.E., Billington N., Ali M.Y., Bookwalter C.S., Lu H., Krementsova E.B. (2018). Recruitment of two dyneins to an mRNA-dependent Bicaudal D transport complex. Elife.

[56] Splinter D., Razafsky D.S., Schlager M.A., Serra-Marques A., Grigoriev I., Demmers J. (2012). BICD2, dynactin, and LIS1 cooperate in regulating dynein recruitment to cellular structures. Mol. Biol. Cell.

[57] Redwine W.B., DeSantis M.E., Hollyer I., Htet Z.M., Tran P.T., Swanson S.K. (2017). The human cytoplasmic dynein interactome reveals novel activators of motility. Elife.

[58] Stevens D.A., Beierschmitt C., Mahesula S., Corley M.R., Salogiannis J., Tsu B.V. (2022). Antiviral function and viral antagonism of the rapidly evolving dynein activating adaptor NINL. Elife.

[59] Bradshaw N.J., Hennah W., Soares D.C. (2013). NDE1 and NDEL1: twin neurodevelopmental proteins with similar ‘nature’ but different ‘nurture’. Biomol. Concepts.

[60] Hebbar S., Mesngon M.T., Guillotte A.M., Desai B., Ayala R., Smith D.S. (2008). Lis1 and Ndel1 influence the timing of nuclear envelope breakdown in neural stem cells. J. Cell Biol..

[61] Wynne C.L., Vallee R.B. (2018). Cdk1 phosphorylation of the dynein adapter Nde1 controls cargo binding from G2 to anaphase. J. Cell Biol..

[62] Yan X., Li F., Liang Y., Shen Y., Zhao X., Huang Q. (2003). Human Nudel and NudE as regulators of cytoplasmic dynein in poleward protein transport along the mitotic spindle. Mol. Cell. Biol..

[63] Bradshaw N.J., Soares D.C., Carlyle B.C., Ogawa F., Davidson-Smith H., Christie S. (2011). PKA phosphorylation of NDE1 is DISC1/PDE4 dependent and modulates its interaction with LIS1 and NDEL1. J. Neurosci..

[64] Klinman E., Holzbaur E.L.F. (2015). Stress-induced CDK5 activation disrupts axonal transport via Lis1/Ndel1/dynein. Cell Rep..

[65] Pandey J.P., Smith D.S. (2011). A Cdk5-dependent switch regulates Lis1/Ndel1/dynein-driven organelle transport in adult axons. J. Neurosci..

[66] Tarricone C., Perrina F., Monzani S., Massimiliano L., Kim M., Derewenda Z. (2004). Coupling PAF signaling to dynein regulation structure of LIS1 in complex with PAF-acetylhydrolase. Neuron.

[67] Feng Y., Olson E.C., Stukenberg P.T., Flanagan L.A., Kirschner M.W., Walsh C.A. (2000). LIS1 regulates CNS lamination by interacting with mNudE, a central component of the centrosome. Neuron.

[68] Soares D.C., Bradshaw N.J., Zou J., Kennaway C.K., Hamilton R.S., Chen Z.A. (2012). The mitosis and neurodevelopment proteins NDE1 and NDEL1 form dimers, tetramers, and polymers with a folded back structure in solution∗. J. Biol. Chem..

[69] Mirdita M., Schütze K., Moriwaki Y., Heo L., Ovchinnikov S., Steinegger M. (2022). ColabFold: making protein folding accessible to all. Nat. Methods.

[70] Cianfrocco M.A., Wong-Barnum M., Youn C., Wagner R., Leschziner A. (2017). COSMIC2: a science gateway for Cryo-electron microscopy structure determination. In Proceedings of the Practice and Experience in Advanced Research Computing 2017 on Sustainability, Success and Impact.

[71] Jumper J., Evans R., Pritzel A., Green T., Figurnov M., Ronneberger O. (2021). Highly accurate protein structure prediction with AlphaFold. Nature.

[72] Reck-Peterson S.L., Yildiz A., Carter A.P., Gennerich A., Zhang N., Vale R.D. (2006). Single-molecule analysis of dynein processivity and stepping behavior. Cell.

[73] Li J., Lee W.-L., Cooper J.A. (2005). NudEL targets dynein to microtubule ends through LIS1. Nat. Cell Biol..

[74] Efimov V.P., Zhang J., Xiang X. (2006). CLIP-170 homologue and NUDE play overlapping roles in NUDF localization in Aspergillus nidulans. Mol. Biol. Cell.

[75] Lara-Gonzalez P., Pines J., Desai A. (2021). Spindle assembly checkpoint activation and silencing at kinetochores. Semin. Cell Dev. Biol..

[76] Lara-Gonzalez P., Westhorpe F.G., Taylor S.S. (2012). The spindle assembly checkpoint. Curr. Biol..

[77] Agrawal R., Gillies J.P., Zang J.L., Zhang J., Garrott S.R., Shibuya H. (2022). The KASH5 protein involved in meiotic chromosomal movements is a novel dynein activating adaptor. Elife.

[78] Pollard T.D. (2010). A guide to simple and informative binding assays. Mol. Biol. Cell.

[79] Case R.B., Pierce D.W., Hom-Booher N., Hart C.L., Vale R.D. (1997). The directional preference of kinesin motors is specified by an element outside of the motor catalytic domain. Cell.

[80] Roberts A.J., Goodman B.S., Reck-Peterson S.L. (2014). Reconstitution of dynein transport to the microtubule plus end by kinesin. Elife.

[81] Ton W.D., Wang Y., Chai P., Beauchamp-Perez C., Flint N.T., Lammers L.G. (2022). Microtubule binding-induced allostery promotes LIS1 dissociation from dynein prior to cargo transport. bioRxiv.

