# Supplementary figures and images for: Ndel1 disfavors dynein–dynactin–adaptor complex formation in two distinct ways
Source: J Biol Chem. 2023 Apr 21;299(6):104735. doi: 10.1016/j.jbc.2023.104735 (PMC10248797; doi:10.1016/j.jbc.2023.104735)

**FIGURE S1**

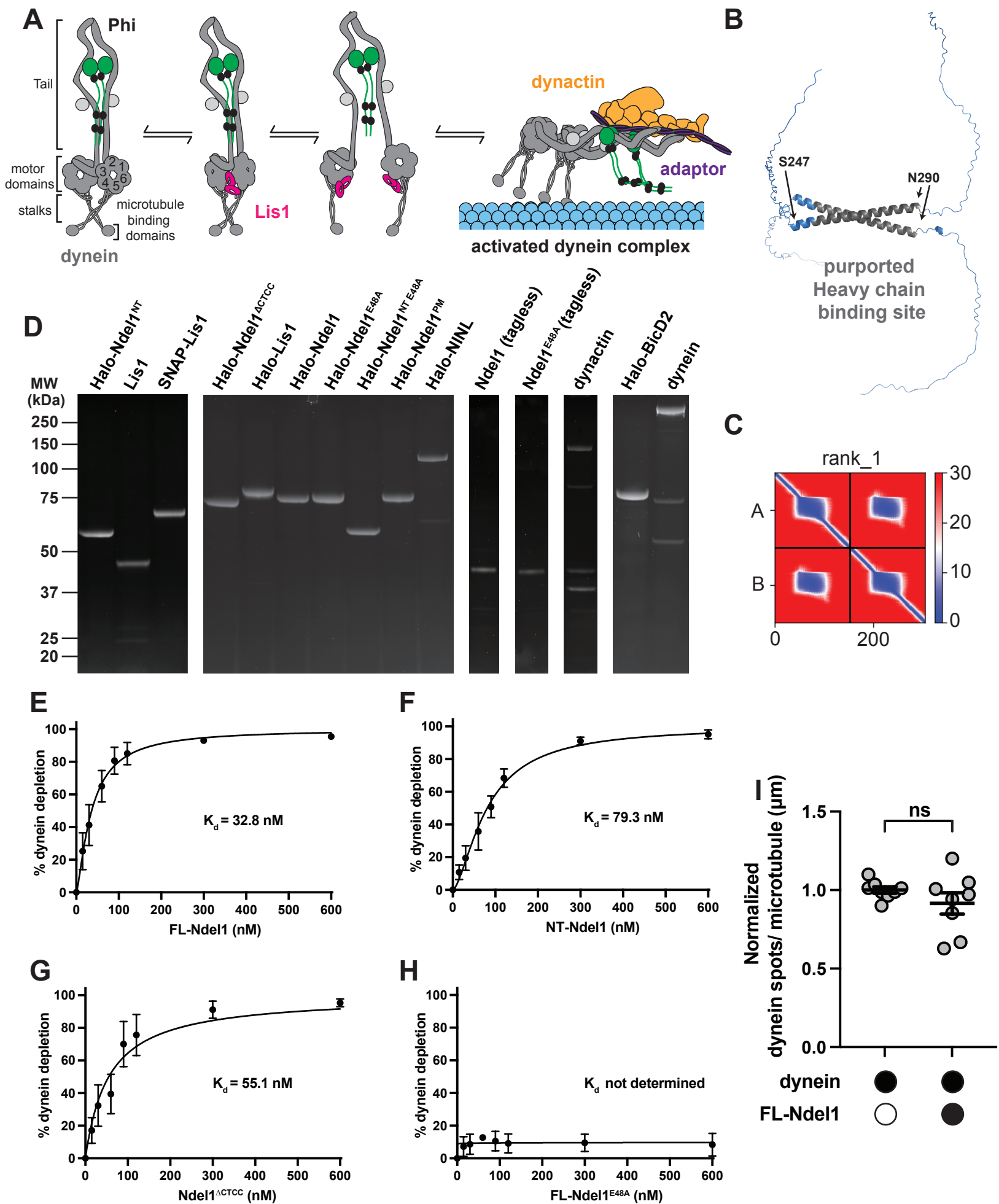

Supplement: Supporting Figure S1 — A, Model of Lis1 favoring open dynein and leading to activated dynein complex formation (16, 17, 19, 23, 24). B, Model of the C terminus of Ndel1 (from amino acids 194–345) generated with AlphaFold showing alpha helical structure. Amino acids located at the beginning and end of the coiled-coil are indicated. Amino acids that have been shown to contribute to heavy chain binding are indicated in grey. C, Predicted alignment error plot for the AlphaFold model in (B). D, SDS-PAGE gels of all purified proteins used in this study. E, Binding curve between dynein and FL-Ndel1. n = 6. Error bars are mean ± SD. F, Binding curve between dynein and NT-Ndel1. n = 4. Error bars are mean ± SD. G, Binding curve between dynein and FL-Ndel1ΔCTCC. n = 3. Error bars are mean ± SD. H, Binding curve between dynein and FL-Ndel1E48A. n = 3. Error bars are mean ± SD. 5 nM dynein was used in each binding experiment. I, Normalized dynein spots/μm in the absence (white circles) and presence (black circles) of 300 nM FL-Ndel1. Each point represents a field of view with 15 to 25 microtubules. n = 8. Error bars are mean ± SEM. Statistical analysis was performed using Welch’s T test. p values: ns = 0.2641. [file mmc1.pdf]

FIGURE S2

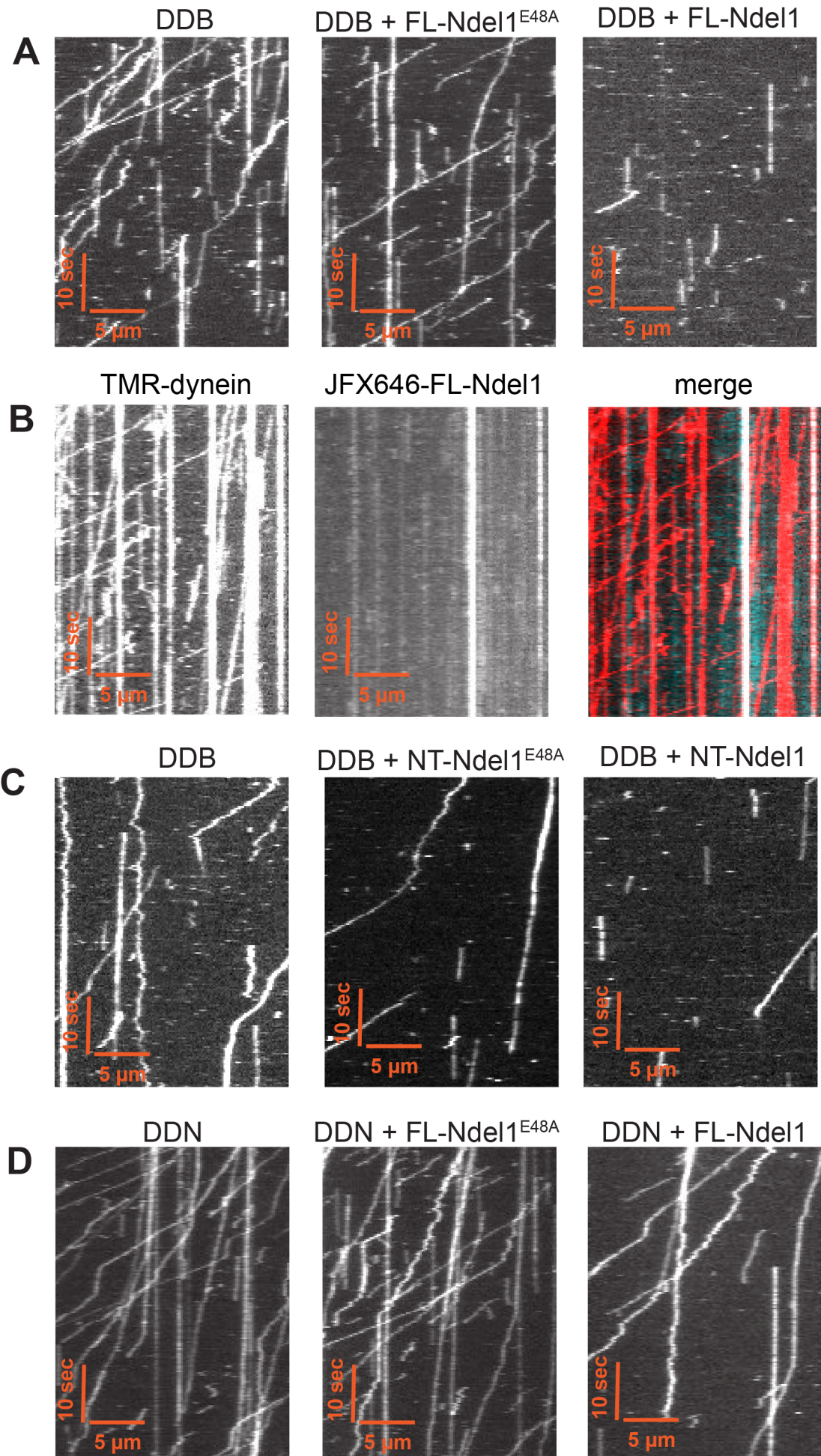

Supplement: Supporting Figure S2 — A, Example kymographs of DDB with and without 300 nM FL-Ndel1E48A and FL-Ndel1. B, Example kymographs of DDB (TMR dynein; red in merge) with 10 nM Janelia Fluor-647 labelled FL-Ndel1 (cyan in merge). C, Example kymographs of DDB with and without 300 nM NT-Ndel1E48A and NT-Ndel1. D, Example kymographs of DDN with and without 300 nM FL-Ndel1E48A and FL-Ndel1. [file mmc2.pdf]

FIGURE S3

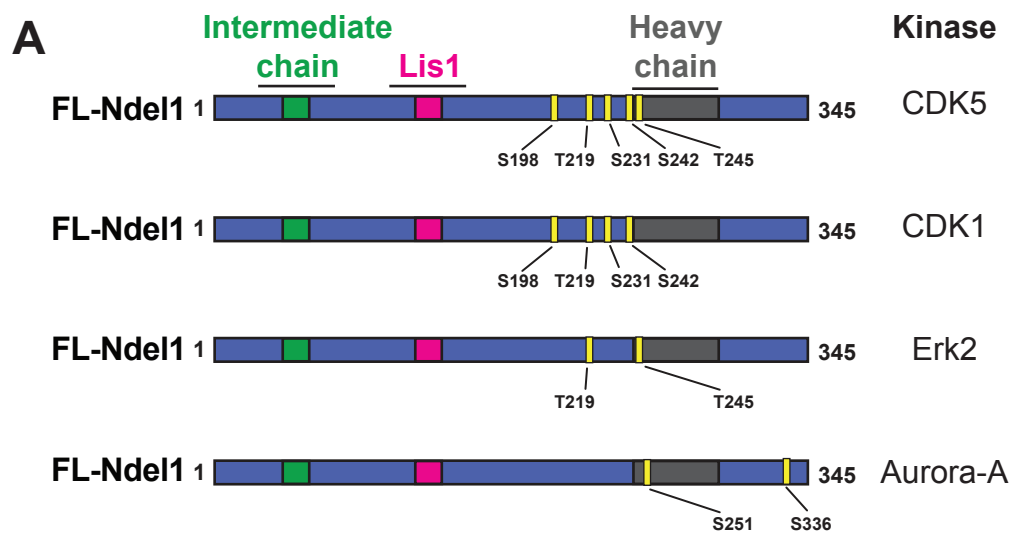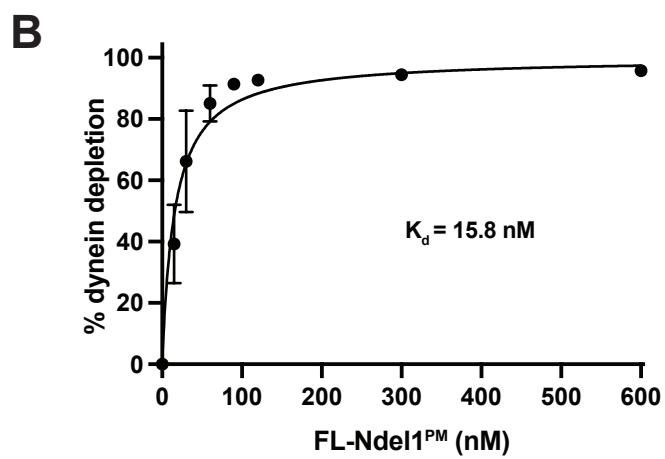

Supplement: Supporting Figure S3 — A, Schematics of FL-Ndel1 showing the residues phosphorylated by CDK5, CDK1, Erk2 and Aurora-A. B, Binding curve between dynein and FL-Ndel1PM. n = 3. Error bars are mean ± SD. [file mmc3.pdf]

FIGURE S4

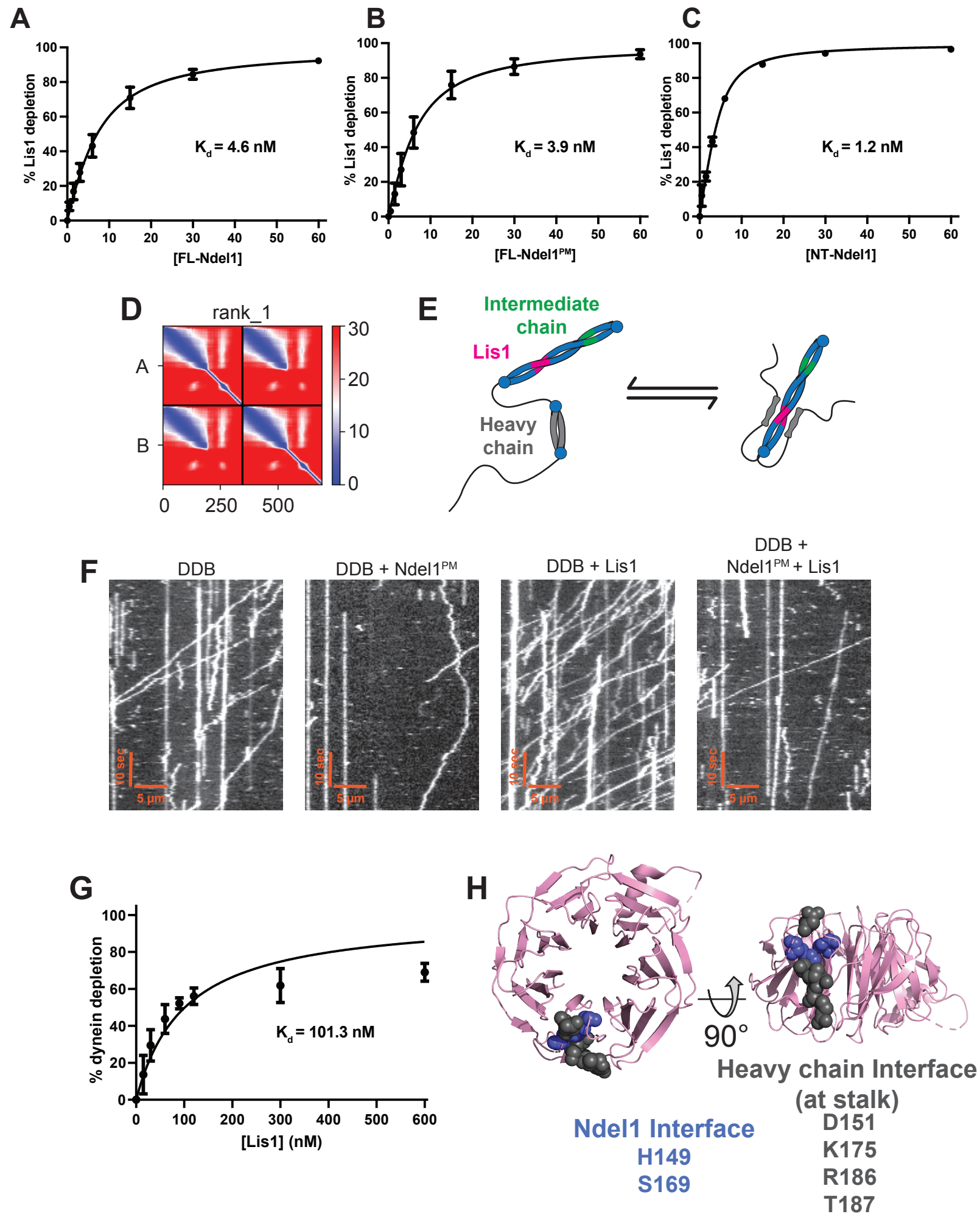

Supplement: Supporting Figure S4 — A, Binding curve between Lis1 and FL-Ndel1. n = 6. Error bars are mean ± SD. B, Binding curve between Lis1 and FL-Ndel1PM. n = 4. Error bars are mean ± SD. C, Binding curve between Lis1 and NT-Ndel1. n = 3. Error bars are mean ± SD. D, Predicted alignment error plot for the AlphaFold model in Figure 4B. E, Conformational equilibrium of FL-Ndel1. F, Example kymographs of DDB with and without 50 nM FL-Ndel1PM and Lis1. G, Binding curve between dynein and Lis1. n = 3. Error bars are mean ± SD. H, Model of the Lis1 beta propeller (pink) (PDB: 1VYH) showing residues known to interact with Ndel1(blue) and dynein’s stalk (gray) (20, 66, 67). [file mmc4.pdf]
